# Effectiveness of a Blended Care Self-Management Program for Caregivers of People With Early-Stage Dementia (Partner in Balance): Randomized Controlled Trial

**DOI:** 10.2196/10017

**Published:** 2018-07-13

**Authors:** Lizzy MM Boots, Marjolein E de Vugt, Gertrudis IJM Kempen, Frans RJ Verhey

**Affiliations:** ^1^ Alzheimer Center Limburg, Department of Psychiatry and Neuropsychology School for Mental Health and Neurosciences Maastricht University Medical Center Maastricht Netherlands; ^2^ Department of Health Services Research Care and Public Health Research Institute Maastricht University Maastricht Netherlands

**Keywords:** internet, caregivers, technology, therapeutics

## Abstract

**Background:**

The benefits of electronic health support for dementia caregivers are increasingly recognized. Reaching caregivers of people with early-stage dementia could prevent high levels of burden and psychological problems in the later stages.

**Objective:**

The current study evaluates the effectiveness of the blended care self-management program, Partner in Balance, compared to a control group.

**Methods:**

A single-blind randomized controlled trial with 81 family caregivers of community-dwelling people with mild dementia was conducted. Participants were randomly assigned to either the 8-week, blended care self-management Partner in Balance program (N=41) or a waiting-list control group (N=40) receiving usual care (low-frequent counseling). The program combines face-to-face coaching with tailored Web-based modules. Data were collected at baseline and after 8 weeks in writing by an independent research assistant who was blinded to the treatment. The primary proximal outcome was self-efficacy (Caregiver Self-Efficacy Scale) and the primary distal outcome was symptoms of depression (Center for Epidemiological Studies Depression Scale). Secondary outcomes included mastery (Pearlin Mastery Scale), quality of life (Investigation Choice Experiments for the Preferences of Older People), and psychological complaints (Hospital Anxiety and Depression Scale-Anxiety and Perceived Stress Scale).

**Results:**

A significant increase in favor of the intervention group was demonstrated for self-efficacy (care management, *P*=.002; service use *P*=.001), mastery (*P*=.001), and quality of life (*P*=.032). Effect sizes were medium for quality of life (*d*=0.58) and high for self-efficacy care management and service use (*d*=0.85 and *d*=0.93, respectively) and mastery (*d*=0.94). No significant differences between the groups were found on depressive symptoms, anxiety, and perceived stress.

**Conclusions:**

This study evaluated the first blended-care intervention for caregivers of people with early-stage dementia and demonstrated a significant improvement in self-efficacy, mastery, and quality of life after receiving the Partner in Balance intervention, compared to a waiting-list control group receiving care as usual. Contrary to our expectations, the intervention did not decrease symptoms of depression, anxiety, or perceived stress. However, the levels of psychological complaints were relatively low in the study sample. Future studies including long-term follow up could clarify if an increase in self-efficacy results in a decrease or prevention of increased stress and depression. To conclude, the program can provide accessible preventative care to future generations of caregivers of people with early-stage dementia.

**Trial Registration:**

Netherlands Trial Register NTR4748; http://www.trialregister.nl/trialreg/admin/rctview.asp?TC=4748 (Archived by WebCite at http://www.webcitation.org/6vSb2t9Mg)

## Introduction

The majority of people with dementia are living at home and cared for by a family member, the informal caregiver. Informal care will be increasingly important as the number of people with dementia has been predicted rise to 65.7 million by 2030 and 115.4 million by 2050, together with a decrease of the working population [1].

However, informal caregiving has a downside. Caregivers of people with dementia are vulnerable due to the chronic stress they experience in the caregiving process [2], which may result in depression, anxiety, and other health problems [3]. Many caregiver support interventions have been developed to ameliorate negative caregiver consequences with promising results [4].

Early intervention and support for caregivers could prevent high levels of burden and psychological problems in the later stages of dementia [5,6]. However, early-stage interventions may not be effective, and even do more harm than good if they do not fit the personal situation of the caregiver. Negative and stigmatizing information can hamper acceptance, while enhancing the positive, intact experiences may be effective in increasing caregiver self-efficacy [7]. The Stress and Coping paradigm by Lazarus and Folkman [8] and the Social Learning theory by Bandura [9] propose that taking charge of the changes in one’s life has a positive effect on self-efficacy and can therefore reduce caregiver stress and its negative impact on general wellbeing [10]. By increasing caregiver resilience through self-efficacy, an increase of psychological problems in a later stage may be prevented [9]. A self-management approach provides an excellent opportunity to actively involve caregivers and let them choose the themes and strategies that are best tailored to their needs. This suits the caring role transition in the early stages, which leans more towards a focus on positively managing life with dementia rather than managing the dementia itself [11].

With the growing gap between the number of people in need of support and available care professionals [12], electronic health (eHealth) interventions could serve as cost-effective alternatives for dementia caregiver support [13], with increased access and extended reach [14-17]. Blending face-to-face guidance with online support increases client-therapist connection and adherence [18,19]. Although eHealth interventions for caregivers have been developed and evaluated, so far most of them are aimed at dementia related problems in an advanced stage of the caregiver career [20,21] and their overall quality of evidence is low [22]. An iterative step-wise approach was employed to develop the blended care self-management internet-based Partner in Balance (PiB) program for caregivers of people with early-stage dementia. The current study evaluated if PiB is superior to a waiting-list control condition as evidenced by improved subjective self-confidence (self-efficacy and mastery), and lower levels of psychological complaints (symptoms of depression, anxiety, and stress) postintervention.

## Methods

### Overview

This randomized controlled trial was carried out between 2014 and 2016 in the Netherlands. The PiB program was compared to a waiting-list control group receiving usual care. Following the waiting-list period participants were offered the opportunity to follow the PiB program. The Medical Ethics Committee of the Maastricht University Medical Center+ (MUMC+) approved this study (#12-4-059) and the study was registered in the Dutch trial register (NTR4748). The study protocol and supporting SPIRIT checklist are available [23].

From September 2014 to December 2015, family caregivers of people with mild dementia of all subtypes (Clinical Dementia Rating, score 0.5-1) [24] were recruited from memory clinics (MUMC+, Elkerliek Hospital Helmond, Catharina Hospital Eindhoven) and ambulatory mental health clinics (Virenze-RIAGG Maastricht, MET ggz Roermond) in the south of the Netherlands. In addition, caregivers were informed about the trial via caregiver support services, and the website of the Dutch Alzheimer Association. Caregivers were included if they had access to the internet at home, had basic computer skills, and provided written informed consent. Potential participants with insufficient cognitive abilities to engage in the online self-management program, who were overburdened or with severe health problems as determined by study staff, or who cared for people with dementia caused by HIV, acquired brain impairment, Down syndrome, chorea associated with Huntington disease, or alcohol abuse were excluded from participation. Inclusion and exclusion was based on the clinical judgment of the referrer, based on their experience with the target group. Both spouses and other caregivers (eg, children) could be included, as long as they met the criteria above and were >18 years. Details on the recruitment procedure are described in the study protocol [23].

### Randomization and Masking

Following the baseline assessment, participants were randomly assigned to either the PiB program or the waiting-list control group receiving usual care by the first author. Assignment was carried out using a computerized random-number generator for block randomization with variable sizes of 4, 6, and 8. An independent research assistant who was blinded to the allocation of the treatment conducted the postintervention assessments. It was not possible to blind the participants because of obvious differences between the interventions in content (PiB is a multicomponent intervention combining psycho-education, movie clips, assignments, and change plans and usual care often consists of psycho-education) and mode of delivery (PiB blends face-to-face contact with online modules and usual care often consists of face-to-face contact only).

### Intervention and Control

#### Experimental Group: Partner in Balance

**Figure 1:**
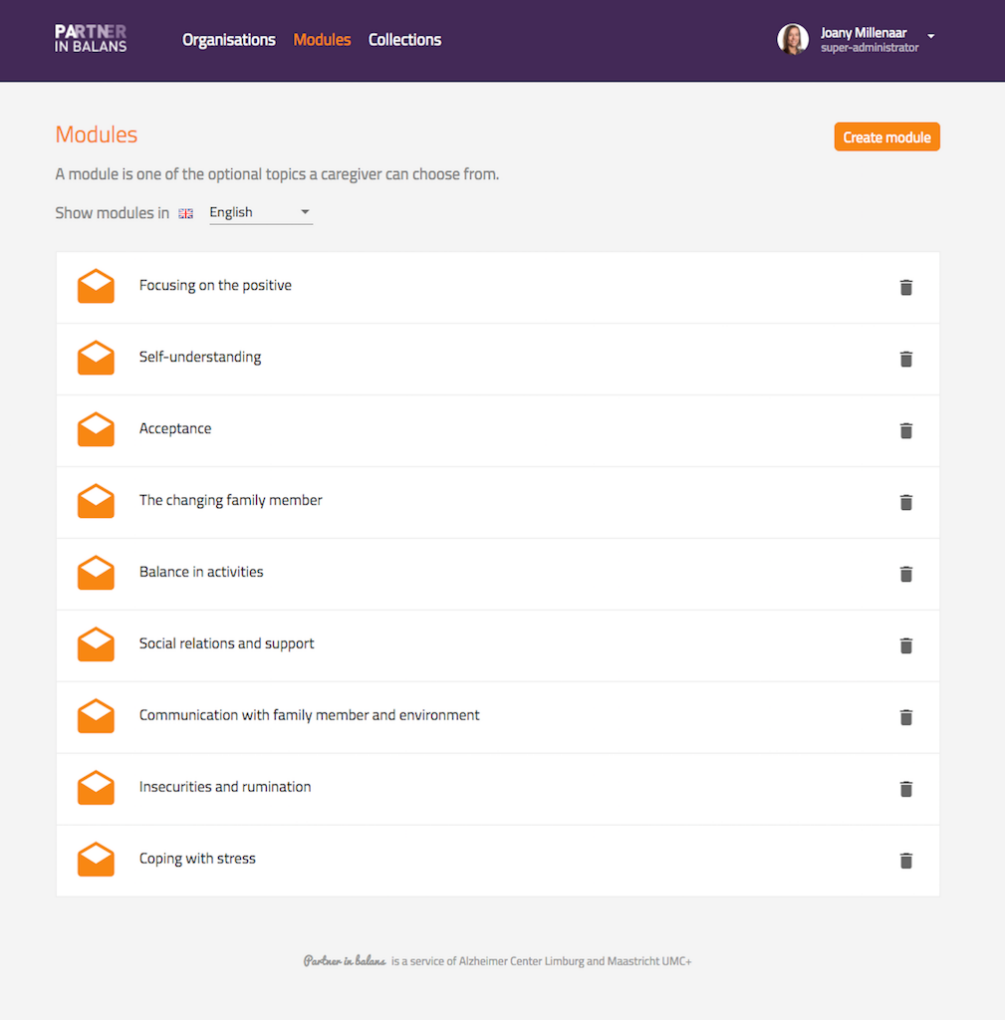
Screenshot of the module themes of the Partner in Balance program.

Detailed information about the program components and development is presented elsewhere [25]. In short, the blended care self-management program PiB consists of: (1) a face-to-face intake session with a personal coach to familiarize participants with the program, set goals, and select preferred module themes; (2) tailored online thematic modules, including psychoeducation, behavioral modeling, reflective assignments, change plans, and email feedback from the coach over 8 weeks; and (3) a face-to-face evaluation session with the coach evaluating previously set goals. All participants in the PiB group received these two face-to-face interactions with the personal coach. Furthermore, the participants can interact with other participants via a discussion forum. Module themes are acceptance, balance in activities, communication with family member and environment, coping with stress, focusing on the positive, insecurities and rumination, self-understanding, the changing family member, and social relations and support. Figure 1 shows a screenshot of the module themes in the program. The participants choose 4 modules and 2 weeks were allocated for each module. However, the participants were allowed to complete the modules at their own pace in accordance with the self-management approach [26]. The personal page and modules remained accessible for participants after the intervention period. The personal coaches were trained, experienced professionals (psychologists and psychiatric nurses) from one of the participating organizations. They attended a 2-hour training session in self-management techniques, goal setting, and online help and attended regular supervision meetings.

**Figure 2:**
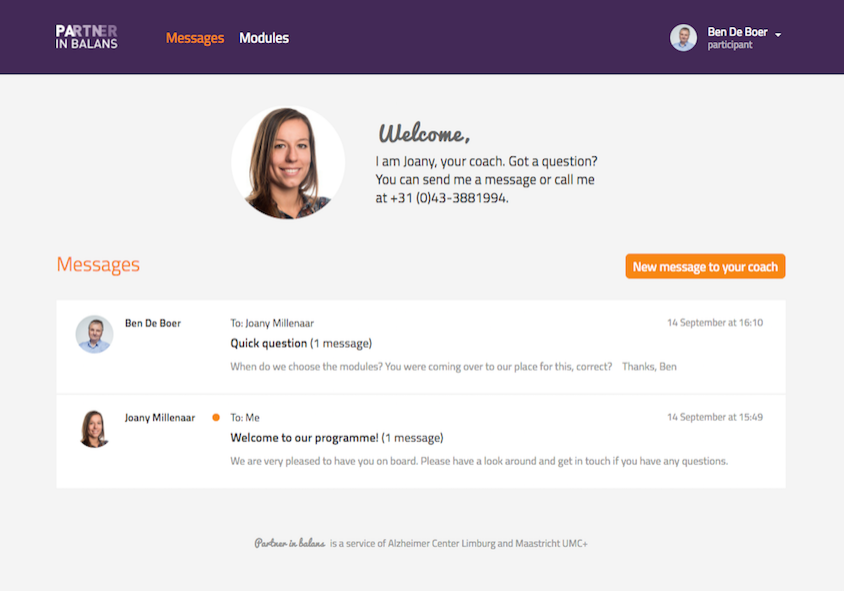
Screenshot of the Partner in Balance program's online messaging portal.

Their tasks were familiarizing participants with the online program, supporting them in module choice and goal setting, and providing feedback on the self-reflective assignments through the online messaging portal in the program (see Figure 2).

#### Control Group: Waiting List

The waiting-list group received usual care consisting of nonfrequent counseling during the 8 weeks. They received the same pretest and posttest attention from the research team as the experimental group. After they completed the posttest assessment, they were given the opportunity to follow PiB.

### Procedures

For this study, self-reported data from the baseline visit (T_0_) and after 8 weeks (T_1_) were compared. These data were collected in writing by an independent research assistant who was blinded to the treatment, separately from the coach visits.

The primary proximal outcome was caregiver self-efficacy and primary distal outcome was depressive symptoms. Caregiver self-efficacy was measured with *The Caregiver Self-Efficacy Scale (CSES)* [27], measuring care management self-efficacy (4 items) and service use self-efficacy (5 items). Care management self-efficacy scores theoretically range from 4-40 and service use self-efficacy from 5-50. Higher scores on the CSES indicate higher levels of self-efficacy. The 20-item *Centre for Epidemiological Studies Depression Scale (CES-D)* [28] was used to measure depressive symptoms. Total scores range from 0-60; where higher scores indicate more symptoms. Secondary outcomes were mastery, psychological complaints (anxiety and perceived stress), and quality of life. Mastery was measured with the 7-item *Pearlin Mastery Scale (PMS)* [29]. The total score ranges from 7-35; where higher scores indicate higher levels of mastery. The 7-item *Hospital and Anxiety Depression Scale-Anxiety (HADS-A)* [30] rates symptoms of anxiety. Scores theoretically range from 0-21 with higher scores indicating more symptoms. Quality of life was measured on five attributes with the *Investigating Choice Experiments for the Preferences of Older People CAPability measure for Older people (ICECAP-O)* [31]. This index value indicates how good or bad the average person aged 65 or older considers a given state to be, for instance attributing to “attachment” (love and friendship) and “control” (independence). The value system for the 1024 (4^5^=1024) possible states uses a best-worst scaling valuation method, providing a single summary score, anchored at zero (“no capability”) and 1.0 (“full capability”) [32].

Demographics were obtained (sex, age, relationship to care recipient, level of education, sharing household, and care intensity in years). The *Global Deterioration Scale* (GDS) [33] measured dementia severity with the caregiver as the informant. The possible modifying effects of the following variables were measures. Quality of the relationship was measured using 4 self-rating items of the University of Southern California Longitudinal Study of Three-Generation Families measures of positive affect [34]. The 12-item Emotional instability domain of the *NEO Five Factory Inventory (NEO-FFI)* [35], was used to identifying individuals who are prone to psychological distress, by assessing 6 traits: anxiety, angry hostility, depression, self-consciousness, impulsiveness, and vulnerability. Scores ranged from 0-24; where higher scorers are likely to be sensitive, emotional, and more prone to experiencing feelings that are upsetting.

### Sample Size

We aimed to enroll 80 participants (40 participants per group), based on previous online intervention studies in caregivers of people with dementia with the CSES as outcome measure, on the basis of repeated measures, within-between interaction with a mean effect size of 0.2 [36], assuming an alpha of .05, a power of 85%, and 25% loss to follow-up.

### Data Analysis

Prior to the analysis, data were checked for missing values, outliers, and normality. Possible differences between the study groups’ baseline characteristics were tested with *t* tests for continuous variables and chi-square tests for categorical variables. Nonparametric tests (eg, Mann-Whitney *U* Wilcoxon test) were used when necessary in case of nonnormality.

To examine the differences between outcomes for the intervention and the waiting-list control group during the intervention period, an analysis of covariance (ANCOVA) was conducted with outcome at post intervention as the dependent variable, intervention (PiB program, waiting-list control group) as the between-subjects variable and per outcome its baseline value, age, sex, emotional instability, quality of the relationship, educational level, and relationship to the care recipient as covariates. If significant, the intergroup effect size was calculated according to Cohen *d*. Effect sizes of 0.2 were considered small, 0.5 considered medium and 0.8 was considered high [37]. IBM SPSS statistics 22.0 for Macintosh was used and all tests of significance were two-tailed with alpha set at .05 and reported mean change.

### User Involvement

As recommended by the Medical Research Council (MRC) Framework, a stepwise approach was adopted to explore potential user needs, followed by a pilot evaluation to test the feasibility of the intervention and the measurement tools prior to the effect evaluation. The iterative development and pilot evaluation of PiB as recommended by the MRC framework is described elsewhere [25]. The burden of the intervention was assessed in a process evaluation. Further, results were disseminated to study participants by means of a newsletter and PhD thesis.

## Results

### Participants

**Figure 3:**
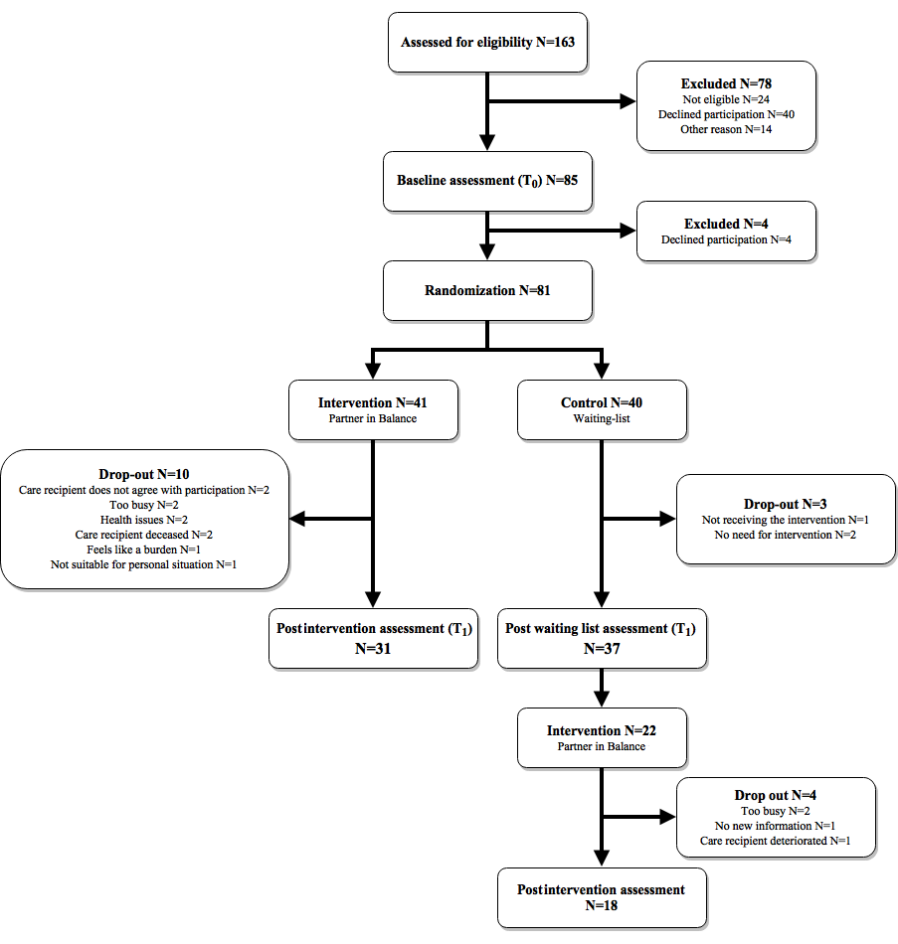
Consolidated Standards of Reporting Trials (CONSORT) study flowchart.

A total of 163 caregivers expressed an interest to participate. See Figure 3 for the study flowchart, the details of which are described elsewhere [38]. Table 1 lists the baseline data for the included caregivers (N=81).

Between-group comparisons revealed no significant differences in demographics and main outcome measures at baseline. Care recipients of the included caregivers were 73.9 years old (SD 8.2), diagnosed with Mild Cognitive Impairment (MCI; 12/81, 15%), Alzheimer’s Disease (AD; 33/81, 41%), or other dementias (36/81, 44%). Dementia severity was rated as preclinical memory decline (55/81, 68%), mild dementia (24/81, 30%), or moderate dementia (2/81, 2%) on the GDS. At T_1_, 13 caregivers were lost to follow-up. The completers did not differ from noncompleters at baseline in terms of age (*t*_79_=0.19; *P*=.851), relationship to the care recipient (χ²_1_=1.39; *P*=.238), same household as care recipient (χ²_1_=0.82; *P*=.665), care intensity in years (*U*=377.5; *P*=.781), sex (χ²_1_=2.80; *P*=.094), education (χ²_1_=1.20; *P*=.550), self-efficacy service use (*t*_79_=0.53; *P*=.599) care management (*t*_79_=1.36; *P*=.177), depression (*U*=280.0; *P*=.266), stress (*t*_79_=0.25, *P*=.806), anxiety (*U*=372.0; *P*=.497), mastery (*t*_79_=–1.18; *P*=.253), and quality of life (*U*=775.0; *P*=.956).

### Intervention Effects

The effects were compared between groups (intervention and waiting-list control) after 8 weeks. Table 2 shows the results of the ANCOVA at T_1_ on self-efficacy (care management and service use), depression, mastery, perceived stress, anxiety, and quality of life. After controlling for age, sex, emotional instability, and quality of the relationship, significant effects in favor of the intervention group were found for self-efficacy care management (*F*_1,60_=10.37; *P*=.002, *d*=0.85), and self-efficacy service use (*F*_1,60_=11.47; *P*=.001; *d*=0.93), but not for depression (*F*_1,60_=1.13; *P*=.293). Significant effects in favor of the intervention group were also demonstrated for mastery (*F*_1,60_=12.66; *P*=.001; *d*=0.94), and quality of life (*F*_1,60_=4.83; *P*=0.032; *d*=0.58), but not for perceived stress (*F*_1,60_=3.40; *P*=0.071), and anxiety (*F*_1,60_=0.80; *P*=.374).

**Table 1:** Descriptive data for caregivers of both groups at baseline.

Demographics and outcome			Intervention (N=41)	Waiting list (N=40)	Test value comparing groups at baseline	*P* value
**Socio-demographics**						
	Age, mean (SD)		67.8 (10.2)	70.2 (10.1)	1.0^a^	.302
	Spouse, n (%)		37 (90.2)	37 (92.5)	0.6^b^	.432
	Same household as PwD^c^, n (%)		39 (95.1)	37 (92.5)	1.3^b^	.513
	Care intensity in years, mean (SD)		1.8 (1.8)	1.9 (1.8)	674.5^d^	.929
	Female, n (%)		29 (70.7)	24 (60.0)	0.8^b^	.385
	**Education, n (%)**					
		High school	8 (19.5)	4 (10.0)	2.3^b^	.321
		College	18 (43.9)	16 (40.0)	—	—
		Graduate school	15 (36.6)	20 (50.0)	—	—
**Primary Outcomes**						
	**Self-efficacy (CSES^e^), mean (SD)**					
		Care management	34.7 (7.8)	33.0 (9.4)	–0.9^a^	.395
		Service use	25.8 (6.3)	23.7 (6.2)	–1.5^a^	.141
	Depression (CES-D^f^), mean (SD)		13.1 (8.7)	13.1 (9.0)	732.0^d^	.927
**Secondary Outcomes**						
	Stress (PSS^g^), mean (SD)		11.8 (6.0)	13.5 (6.2)	1.2^a^	.223
	Anxiety (HADS-A^h^), mean (SD)		6.0 (3.7)	6.7 (4.7)	717.5^d^	.666
	Mastery (PMS^i^), mean (SD)		23.7 (4.1)	22.9 (4.4)	–0.8^a^	.430
	Quality of life (ICECAP-O^j^), mean (SD)		0.8 (0.1)	0.8 (0.1)	755.0^d^	.956

^a^Refers to *t* test (*t*_79_).

^b^Refers to Chi-square test (χ²_1_).

^c^PwD: person with dementia.

^d^Refers to Mann-Whitney *U* Wilcoxon test.

^e^CSES: Caregiver Self-Efficacy Scale.

^f^CES-D: Center for Epidemiological Studies Depression Scale.

^g^PSS: Perceived Stress Scale.

^h^HADS-A: Hospital Anxiety and Depression Scale-Anxiety.

^i^PMS: Pearlin Mastery Scale.

^j^ICECAP-O: Investigating Choice Experiments for the Preferences of Older People.

**Table 2:** Analysis of covariance comparing intervention (N=31) and control (N=37) group at posttest.

Outcome			Control, mean (SD)		Intervention, mean SD		Mean difference^b^ (95% CI)	*F* test (*df*)	Cohen *d*
			Crude^a^	Adjusted^b^	Crude	Adjusted			
**Primary outcomes**									
	**Self-efficacy (CSES^c)^**								
		Care management	31.38 (8.71)	31.65 (1.05)	37.03 (6.33)	36.73 (1.12)	–5.07 (–8.23 to –1.92)	10.37^d^ (1,60)	0.85
		Service use	21.88 (6.33)	22.48 (0.83)	27.43 (5.11)	26.76 (0.89)	–4.27 (–6.80 to –1.75)	11.47^d^ (1,60)	0.93
	Depression (CES-D^e^)		13.27 (9.21)	12.87 (1.08)	10.73 (8.20)	11.17 (1.14)	1.70 (–1.51 to 4.91)	1.13 (1,60)	0.28
**Secondary outcomes**									
	Mastery (PMS^f^)		21.15 (4.49)	21.32 (0.63)	24.87 (4.09)	24.68 (0.67)	–3.36 (–5.26 to –1.47)	12.66 (1,60)^d^	0.94
	Stress (PSS^g^)		13.76 (6.84)	12.92 (0.69)	10.03 (6.35)	10.99 (0.74)	1.94 (–0.17 to 4.04)	3.40 (1,60)	0.50
	Anxiety (HADS-A^h^)		5.94 (4.59)	5.91 (0.61)	6.70 (4.65)	6.73 (0.63)	–0.81 (–2.63 to 1.00)	0.80 (1,60)	0.24
	Quality of life (ICECAP-O^i^)		0.76 (0.15)	0.76 (0.02)	0.82 (0.10)	0.83 (0.02)	–0.06 (–0.12 to –0.01)	4.83 (1,60)^j^	0.58

^a^Group means.

^b^Adjusted for outcome measure at baseline, age, sex, education, quality of the relationship at baseline, neurotic personality traits, and coach background.

^c^CSES: Caregiver Self-Efficacy Scale.

^d^*P*<.01

^e^CES-D: Center for Epidemiological Studies Depression Scale.

^f^PMS: Pearlin Mastery Scale.

^g^PSS: Perceived Stress Scale.

^h^HADS-A: Hospital Anxiety and Depression Scale-Anxiety.

^i^ICECAP-O: Investigating Choice Experiments for the Preferences of Older People.

^j^*P*<.05

## Discussion

### Principal Findings

This randomized controlled study evaluated the first blended-care intervention for caregivers of people with early-stage dementia developed together with potential users, following the MRC Framework, and demonstrated a significant improvement in care management self-efficacy, service use self-efficacy, mastery, and quality of life after receiving the PiB intervention; compared to a waiting-list control group receiving care as usual. Effect sizes were medium (>0.5) for quality of life to high (>0.8) for self-efficacy and mastery. No differences between groups were demonstrated for caregiver depression, anxiety, and perceived stress.

Results on caregiver self-efficacy, mastery, and quality of life are in line with previous results in an uncontrolled study [25] and results of previous eHealth interventions for dementia caregivers [22]. Furthermore, the results of the present study fit the Stress and Coping paradigm by Lazarus and Folkman [8] and the Social Learning theory by Bandura [9], suggesting that taking charge of the changes in one’s life increases self-efficacy and general wellbeing. Learning to positively manage life with dementia instead of managing the dementia itself in a self-management program may have facilitated caregivers’ adaptation to their new caregiving role. The program’s focus on enhancing positive, intact experiences that are tailored to the individual caregiver’s situation could explain the positive effects on caregiver self-efficacy [11]. In addition, the relationship between the participant and the coach may have influenced the outcomes. The process evaluation of the present study showed that both participants and coaches mentioned that their relationship with each other had deepened [38], which was also demonstrated in a previous blended-care intervention for depression [18]. The opportunity to reflect on one’s feelings anonymously in one’s personal safe environment is easier than face-to-face, but the face-to-face contact increased caregiver openness, and therefore coach empathy with their situation [19]. However, we expected that higher levels of wellbeing or quality of life could be the result of a decrease in stress [8,9], which could not be derived from the results of the present study. It is conceivable that interventions aimed at the early stages may not be capable to decrease burden and stress, as these are relatively low during the early stages [7], leaving little room for improvement. Previous caregiver interventions demonstrating positive effects on burden and stress were not specifically aimed at early-stages of dementia [20,39-41]. The process evaluation also revealed that the intervention period and dose varied between participants. Moreover, the discussion forum was not used because caregivers mentioned that sharing their story felt like a betrayal to the care recipient and reading about other people’s “misery” was considered undesirable [38]. These process characteristics may have influenced the intervention effectiveness [42]. Future follow up of PiB effects could clarify if an increase in self-efficacy results in a decrease or prevention of increased stress and depression on the long term.

### Strengths and Limitations

High face validity was demonstrated as the program was evaluated in multiple institutions with multiple coaches of different backgrounds. Development together with the potential users and a pilot evaluation following the MRC Framework may have increased its effectiveness.

The waiting-list period may have affected the differences in outcomes between both groups. The effects of waiting are highly variable and depend on the characteristics of the sample and of the trial [43]. However, this design allowed all potentially interested participants to participate in the intervention program, which may have increased their motivation to participate given that usual care for mild dementia caregivers often either does not include counseling or includes only infrequent counseling [44]. Furthermore, the waiting-list group was not deprived of usual care. An alternative would be a pseudo-intervention in which only psycho-education or only attention of the coach is provided, but the aim of this study was not to evaluate merely the online aspect of the intervention, but the effect of the blended-care intervention of which psycho-education and face-to-face contacts are integral parts.

Intention-to-treat analyses was not fully possible, as intervention noncompleters refused to participate in further assessments. However, we did include participants that were not completely compliant (completed only 2, 3 or no modules at all) in the analyses [38]. Drop-out was higher in the intervention group compared to the control group, which could have resulted in inflated effect sizes. However, selective drop-out was not demonstrated as completers did not differ from noncompleters at baseline. Often mentioned reasons for drop-out were no need for help or refusal by the care recipient, which was demonstrated previously as reasons of nonuse of formal services [45,46]. Furthermore, a higher rate of drop-out in the intervention group has previously been reported. Previous randomized controlled trials even controlled for any possible loss of power beforehand by increasing the sample of the intervention group. Nevertheless, the current effect sizes should be interpreted with caution. Although the power of our group was not jeopardized based on our power calculation, future studies could consider controlling for a higher rate of drop-out in the intervention group to prevent loss of power.

Our sample was not limited to memory clinics only, but the included participants may represent a subgroup of all dementia caregivers in the early stages. Caregivers in the early stages often decline formal care and it is conceivable that many were not familiar with the care parties involved in recruitment and were therefore overlooked in this study [45,46]. This could have resulted in a highly motivated sample more open to support [47]. Furthermore, only computer-literate caregivers could be included, which represents only around 59% of dementia caregivers [48]. However, seniors’ use of internet is expected to rise in the near future [49], increasing the accessibility of PiB.

### Future Research and Clinical Implications

Future research could consider combining all resources used during the intervention period with the intervention costs and outcomes in a cost-consequence analysis to aid decision makers. Furthermore, future research should evaluate sustainability of improvements at long-term follow-up. The higher rate of drop-out in the intervention group showed that this group feels overwhelmed but is perhaps most in need of the intervention. Some eHealth interventions show dropout rates of up to 80% [50-52] and therefore suggest blending face-to-face contacts with online modules, like the PiB program, to prevent these high drop-out rates. We found a relatively high response and participation rate [18], indicating that there is a need for at least having the option to choose for this type of caregiver support.

### Conclusion

In conclusion, this study showed that a blended care self-management program for dementia caregivers in the early stages is effective in increasing caregiver self-efficacy, mastery, and quality of life on the short-term. The program could provide accessible care to future generations of caregivers of people with early-stage dementia and strengthen the primary caregivers.

## Appendix

Multimedia Appendix 1 CONSORT‐EHEALTH checklist (V 1.6.1).

## Abbreviations

ANCOVA: analysis of covariance
CES-D: Center for Epidemiological Studies Depression Scale
CSES: Caregiver Self-Efficacy Scale
eHealth: electronic health
HADS-A: Hospital Anxiety and Depression Scale-Anxiety
ICECAP-O: Investigating Choice Experiments for the Preferences of Older People
MRC: Medical Research Council
MUMC+: Maastricht University Medical Center
PiB: Partner in Balance
PMS: Pearlin Mastery Scale
PSS: Perceived Stress Scale
PwD: person with dementia
