# Supplementary material for: Effectiveness of a Blended Care Self-Management Program for Caregivers of People With Early-Stage Dementia (Partner in Balance): Randomized Controlled Trial
Source: J Med Internet Res. 2018 Jul 13;20(7):e10017. doi: 10.2196/10017 (PMC6064039; doi:10.2196/10017)
Supplement: Multimedia Appendix 1 [file jmir_v20i7e10017_app1.pdf]

# CONSORT-EHEALTH (V 1.6.1) - Submission/Publication Form

The CONSORT-EHEALTH checklist is intended for authors of randomized trials evaluating web-based and Internet-based applications/interventions, including mobile interventions, electronic games (incl multiplayer games), social media, certain telehealth applications, and other interactive and/or networked electronic applications. Some of the items (e.g. all subitems under item 5 - description of the intervention) may also be applicable for other study designs.

The goal of the CONSORT EHEALTH checklist and guideline is to be
a) a guide for reporting for authors of RCTs,
b) to form a basis for appraisal of an ehealth trial (in terms of validity)

CONSORT-EHEALTH items/subitems are MANDATORY reporting items for studies published in the Journal of Medical Internet Research and other journals / scientific societies endorsing the checklist.

Items numbered 1., 2., 3., 4a., 4b etc are original CONSORT or CONSORT-NPT (non-pharmacologic treatment) items.
Items with Roman numerals (i., ii, iii, iv etc.) are CONSORT-EHEALTH extensions/clarifications.

As the CONSORT-EHEALTH checklist is still considered in a formative stage, we would ask that you also RATE ON A SCALE OF 1-5 how important/useful you feel each item is FOR THE PURPOSE OF THE CHECKLIST and reporting guideline (optional).

Mandatory reporting items are marked with a red *.
In the textboxes, either copy & paste the relevant sections from your manuscript into this form - please include any quotes from your manuscript in QUOTATION MARKS,
or answer directly by providing additional information not in the manuscript, or elaborating on why the item was not relevant for this study.

YOUR ANSWERS WILL BE PUBLISHED AS  A SUPPLEMENTARY FILE TO YOUR PUBLICATION IN JMIR AND ARE CONSIDERED PART OF YOUR PUBLICATION (IF ACCEPTED).
Please fill in these questions diligently. Information will not be copyedited, so please use proper spelling and grammar, use correct capitalization, and avoid abbreviations.

DO NOT FORGET TO SAVE AS PDF _AND_ CLICK THE SUBMIT BUTTON SO YOUR ANSWERS ARE IN OUR DATABASE !!!

Citation Suggestion (if you append the pdf as Appendix we suggest to cite this paper in the caption):
Eysenbach G, CONSORT-EHEALTH Group
CONSORT-EHEALTH: Improving and Standardizing Evaluation Reports of Web-based and Mobile Health Interventions
J Med Internet Res 2011;13(4):e126
URL: http://www.jmir.org/2011/4/e126/
doi: 10.2196/jmir.1923
PMID: 22209829

* Required

## Your name *

First Last

Lizzy Boots

## Primary Affiliation (short), City, Country *

University of Toronto, Toronto, Canada

Maastricht University, Maastricht, the Ne

## Your e-mail address *

abc@gmail.com

l.boots@maastrichtuniversity.nl

## Title of your manuscript *

Provide the (draft) title of your manuscript.

Effectiveness of the blended care self-management programme "Partner in Balance" for early-stage dementia caregivers: results of a randomized controlled trial

## Name of your App/Software/Intervention *

If there is a short and a long/alternate name, write the short name first and add the long name in brackets.

Partner in Balance

## Evaluated Version (if any)

e.g. "V1", "Release 2017-03-01", "Version 2.0.27913"

Your answer

## Language(s) *

What language is the intervention/app in? If multiple languages are available, separate by comma (e.g. "English, French")

Dutch

## URL of your Intervention Website or App *

e.g. a direct link to the mobile app on app in appstore (itunes, Google Play), or URL of the website. If the intervention is a DVD or hardware, you can also link to an Amazon page.

http://www.partnerinbalans.nl

## URL of an image/screenshot (optional)

Your answer

## Accessibility *

Can an enduser access the intervention presently?

- ○ access is free and open
- ● access only for special usergroups, not open
- ○ access is open to everyone, but requires payment/subscription/in-app purchases
- ○ app/intervention no longer accessible
- ○ Other:

## Primary Medical Indication/Disease/Condition *

e.g. "Stress", "Diabetes", or define the target group in brackets after the condition, e.g. "Autism (Parents of children with)", "Alzheimers (Informal Caregivers of)"

Dementia (informal caregivers of)

## Primary Outcomes measured in trial *

comma-separated list of primary outcomes reported in the trial

self-efficacy, depression

## Secondary/other outcomes

Are there any other outcomes the intervention is expected to affect?

mastery, quality of life, psychological complaints (anxiety and perceived stress)

## Recommended "Dose" *

What do the instructions for users say on how often the app should be used?

- ○ Approximately Daily
- ◉ Approximately Weekly
- ○ Approximately Monthly
- ○ Approximately Yearly
- ○ "as needed"
- ○ Other:

## Approx. Percentage of Users (starters) still using the app as recommended after 3 months *

- ○ unknown / not evaluated
- ○ 0-10%
- ○ 11-20%
- ○ 21-30%
- ○ 31-40%
- ○ 41-50%
- ○ 51-60%
- ○ 61-70%
- ○ 71%-80%
- ◉ 81-90%
- ○ 91-100%
- ○ Other:

## Overall, was the app/intervention effective? *

- ○ yes: all primary outcomes were significantly better in intervention group vs control
- ◉ partly: SOME primary outcomes were significantly better in intervention group vs control
- ○ no statistically significant difference between control and intervention
- ○ potentially harmful: control was significantly better than intervention in one or more outcomes
- ○ inconclusive: more research is needed
- ○ Other:

## Article Preparation Status/Stage *

At which stage in your article preparation are you currently (at the time you fill in this form)

- ○ not submitted yet - in early draft status
- ◉ not submitted yet - in late draft status, just before submission
- ○ submitted to a journal but not reviewed yet
- ○ submitted to a journal and after receiving initial reviewer comments
- ○ submitted to a journal and accepted, but not published yet
- ○ published
- ○ Other:

## Journal *

If you already know where you will submit this paper (or if it is already submitted), please provide the journal name (if it is not JMIR, provide the journal name under "other")

- ○ not submitted yet / unclear where I will submit this
- ◉ Journal of Medical Internet Research (JMIR)
- ○ JMIR mHealth and UHealth
- ○ JMIR Serious Games
- ○ JMIR Mental Health
- ○ JMIR Public Health
- ○ JMIR Formative Research
- ○ Other JMIR sister journal
- ○ Other:

## Is this a full powered effectiveness trial or a pilot/feasibility trial? *

- ○ Pilot/feasibility
- ◉ Fully powered

## Manuscript tracking number *

If this is a JMIR submission, please provide the manuscript tracking number under "other" (The ms tracking number can be found in the submission acknowledgement email, or when you login as author in JMIR. If the paper is already published in JMIR, then the ms tracking number is the four-digit number at the end of the DOI, to be found at the bottom of each published article in JMIR)

- ◉ no ms number (yet) / not (yet) submitted to / published in JMIR
- ○ Other:

# TITLE AND ABSTRACT

## 1a) TITLE: Identification as a randomized trial in the title

### 1a) Does your paper address CONSORT item 1a? *

I.e does the title contain the phrase "Randomized Controlled Trial"? (if not, explain the reason under "other")

- (●) yes
- ( ) Other:

### 1a-i) Identify the mode of delivery in the title

Identify the mode of delivery. Preferably use "web-based" and/or "mobile" and/or "electronic game" in the title. Avoid ambiguous terms like "online", "virtual", "interactive". Use "Internet-based" only if Intervention includes non-web-based Internet components (e.g. email), use "computer-based" or "electronic" only if offline products are used. Use "virtual" only in the context of "virtual reality" (3-D worlds). Use "online" only in the context of "online support groups". Complement or substitute product names with broader terms for the class of products (such as "mobile" or "smart phone" instead of "iphone"), especially if the application runs on different platforms.

| | 1 | 2 | 3 | 4 | 5 | |
|---|---|---|---|---|---|---|
| subitem not at all important | ( ) | ( ) | ( ) | (●) | ( ) | essential |

### Does your paper address subitem 1a-i? *

Copy and paste relevant sections from manuscript title (include quotes in quotation marks "like this" to indicate direct quotes from your manuscript), or elaborate on this item by providing additional information not in the ms, or briefly explain why the item is not applicable/relevant for your study

"Effectiveness of the blended care self-management program "Partner in Balance" for early-stage dementia caregivers: results of a randomized controlled trial"

Blended care means the blending of web-based and face-to-face care. This intervention is delivered in a blended format.

## 1a-ii) Non-web-based components or important co-interventions in title

Mention non-web-based components or important co-interventions in title, if any (e.g., “with telephone support”).

| | 1 | 2 | 3 | 4 | 5 | |
|---|---|---|---|---|---|---|
| subitem not at all important | ○ | ○ | ○ | ◉ | ○ | essential |

## Does your paper address subitem 1a-ii?

Copy and paste relevant sections from manuscript title (include quotes in quotation marks "like this" to indicate direct quotes from your manuscript), or elaborate on this item by providing additional information not in the ms, or briefly explain why the item is not applicable/relevant for your study

"Effectiveness of the blended care self-management program “Partner in Balance” for early-stage dementia caregivers: results of a randomized controlled trial"

Blended care means the blending of web-based and face-to-face care. This intervention is delivered in a blended format.

## 1a-iii) Primary condition or target group in the title

Mention primary condition or target group in the title, if any (e.g., “for children with Type I Diabetes”)
Example: A Web-based and Mobile Intervention with Telephone Support for Children with Type I Diabetes: Randomized Controlled Trial

| | 1 | 2 | 3 | 4 | 5 | |
|---|---|---|---|---|---|---|
| subitem not at all important | ○ | ○ | ○ | ○ | ◉ | essential |

## Does your paper address subitem 1a-iii? *

Copy and paste relevant sections from manuscript title (include quotes in quotation marks "like this" to indicate direct quotes from your manuscript), or elaborate on this item by providing additional information not in the ms, or briefly explain why the item is not applicable/relevant for your study

"Effectiveness of the blended care self-management program “Partner in Balance” for early-stage dementia caregivers: results of a randomized controlled trial"

The intervention was developed for early-stage dementia caregivers

## 1b) ABSTRACT: Structured summary of trial design, methods, results, and conclusions

NPT extension: Description of experimental treatment, comparator, care providers, centers, and blinding status.

## 1b-i) Key features/functionalities/components of the intervention and comparator in the METHODS section of the ABSTRACT

Mention key features/functionalities/components of the intervention and comparator in the abstract. If possible, also mention theories and principles used for designing the site. Keep in mind the needs of systematic reviewers and indexers by including important synonyms. (Note: Only report in the abstract what the main paper is reporting. If this information is missing from the main body of text, consider adding it)

| | 1 | 2 | 3 | 4 | 5 | |
|---|---|---|---|---|---|---|
| subitem not at all important | ○ | ○ | ○ | ○ | ◉ | essential |

## Does your paper address subitem 1b-i? *

Copy and paste relevant sections from the manuscript abstract (include quotes in quotation marks "like this" to indicate direct quotes from your manuscript), or elaborate on this item by providing additional information not in the ms, or briefly explain why the item is not applicable/relevant for your study

"The benefits of e-health support for dementia caregivers are increasingly recognised. Reaching early-stage dementia caregivers could prevent high levels of burden and psychological problems in the later stages.

"Participants were randomly assigned to either the 8-week blended care self-management program PiB or a waiting-list control group receiving usual care (low-frequent counselling). PiB combines face-to-face coaching with tailored web-based modules."

## 1b-ii) Level of human involvement in the METHODS section of the ABSTRACT

Clarify the level of human involvement in the abstract, e.g., use phrases like “fully automated” vs. “therapist/nurse/care provider/physician-assisted” (mention number and expertise of providers involved, if any). (Note: Only report in the abstract what the main paper is reporting. If this information is missing from the main body of text, consider adding it)

| | 1 | 2 | 3 | 4 | 5 | |
|---|---|---|---|---|---|---|
| subitem not at all important | ○ | ○ | ○ | ◉ | ○ | essential |

## Does your paper address subitem 1b-ii?

Copy and paste relevant sections from the manuscript abstract (include quotes in quotation marks "like this" to indicate direct quotes from your manuscript), or elaborate on this item by providing additional information not in the ms, or briefly explain why the item is not applicable/relevant for your study

"blended care self-management program ‘Partner in Balance’ or a waiting-list control group receiving usual care (low-frequent counselling)."

"Participants were randomly assigned to either the 8-week blended care self-management program PiB or a waiting-list control group receiving usual care (low-frequent counselling). PiB combines face-to-face coaching with tailored web-based modules."

Blended care means the blending of web-based and face-to-face care. Personal coaches perform the face-to-face and online guidance, which is further explained in the methods section of the paper.
In the process paper which was published in JMIR recently (#ms 7666, PMID: 29258980) the abstract elaborates on the personal coaches and their background. To avoid overlap, this was not the focus of the current effect paper.

## 1b-iii) Open vs. closed, web-based (self-assessment) vs. face-to-face assessments in the METHODS section of the ABSTRACT

Mention how participants were recruited (online vs. offline), e.g., from an open access website or from a clinic or a closed online user group (closed usergroup trial), and clarify if this was a purely web-based trial, or there were face-to-face components (as part of the intervention or for assessment). Clearly say if outcomes were self-assessed through questionnaires (as common in web-based trials). Note: In traditional offline trials, an open trial (open-label trial) is a type of clinical trial in which both the researchers and participants know which treatment is being administered. To avoid confusion, use “blinded” or “unblinded” to indicated the level of blinding instead of “open”, as “open” in web-based trials usually refers to “open access” (i.e. participants can self-enrol). (Note: Only report in the abstract what the main paper is reporting. If this information is missing from the main body of text, consider adding it)

| | 1 | 2 | 3 | 4 | 5 | |
|---|---|---|---|---|---|---|
| subitem not at all important | ○ | ○ | ○ | ○ | ◉ | essential |

## Does your paper address subitem 1b-iii?

Copy and paste relevant sections from the manuscript abstract (include quotes in quotation marks "like this" to indicate direct quotes from your manuscript), or elaborate on this item by providing additional information not in the ms, or briefly explain why the item is not applicable/relevant for your study

(Quality and procedure of) recruitment is described in detail in the recently published process paper (JMIR MS #7666; PMID: 29258980).

Assessment: "Data were collected at baseline and after 8 weeks in writing by an independent research assistant who was unknown to the allocation of the treatment."

## 1b-iv) RESULTS section in abstract must contain use data

Report number of participants enrolled/assessed in each group, the use/uptake of the intervention (e.g., attrition/adherence metrics, use over time, number of logins etc.), in addition to primary/secondary outcomes. (Note: Only report in the abstract what the main paper is reporting. If this information is missing from the main body of text, consider adding it)

| | 1 | 2 | 3 | 4 | 5 | |
|---|---|---|---|---|---|---|
| subitem not at all important | ○ | ○ | ○ | ○ | ◉ | essential |

## Does your paper address subitem 1b-iv?

Copy and paste relevant sections from the manuscript abstract (include quotes in quotation marks "like this" to indicate direct quotes from your manuscript), or elaborate on this item by providing additional information not in the ms, or briefly explain why the item is not applicable/relevant for your study

"Participants were randomly assigned to either the 8-week blended care self-management program PiB (N=41) or a waiting-list control group (N=40) receiving usual care (low-frequent counselling)."

Use/uptake of the intervention and details are described in the recently published process paper (JMIR MS #7666; PMID: 29258980)

## 1b-v) CONCLUSIONS/DISCUSSION in abstract for negative trials

Conclusions/Discussions in abstract for negative trials: Discuss the primary outcome - if the trial is negative (primary outcome not changed), and the intervention was not used, discuss whether negative results are attributable to lack of uptake and discuss reasons. (Note: Only report in the abstract what the main paper is reporting. If this information is missing from the main body of text, consider adding it)

| | 1 | 2 | 3 | 4 | 5 | |
|---|---|---|---|---|---|---|
| subitem not at all important | ○ | ○ | ○ | ○ | ◉ | essential |

## Does your paper address subitem 1b-v?

Copy and paste relevant sections from the manuscript abstract (include quotes in quotation marks "like this" to indicate direct quotes from your manuscript), or elaborate on this item by providing additional information not in the ms, or briefly explain why the item is not applicable/relevant for your study

"A significant increase in favor of the intervention group was demonstrated for self-efficacy (care management P=0.002, service use P=0.001), mastery (P=0.001), and quality of life (P=0.032). Effect sizes were medium for quality of life (d=0.58) to high for self-efficacy (d=0.85 and d=0.94, respectively) and mastery (d=0.94). No significant differences between the groups were found on depressive symptoms, anxiety and perceived stress."

"Contrary to our expectations, the intervention did not decrease symptoms of depression and anxiety and perceived stress. However, levels of psychological complaints were relatively low in the study sample."

Further details on the intervention uptake and possible implications for the intervention effectiveness are described in detail in the recently published process paper (JMIR MS #7666; PMID: 29258980)

## INTRODUCTION

## 2a) In INTRODUCTION: Scientific background and explanation of rationale

## 2a-i) Problem and the type of system/solution

Describe the problem and the type of system/solution that is object of the study: intended as stand-alone intervention vs. incorporated in broader health care program? Intended for a particular patient population? Goals of the intervention, e.g., being more cost-effective to other interventions, replace or complement other solutions? (Note: Details about the intervention are provided in “Methods” under 5)

| | 1 | 2 | 3 | 4 | 5 | |
|---|---|---|---|---|---|---|
| subitem not at all important | ○ | ○ | ○ | ○ | ◉ | essential |

## Does your paper address subitem 2a-i? *

Copy and paste relevant sections from the manuscript (include quotes in quotation marks "like this" to indicate direct quotes from your manuscript), or elaborate on this item by providing additional information not in the ms, or briefly explain why the item is not applicable/relevant for your study

Family caregivers are important as the main source of care for people with dementia, but caregiving puts them at risk for feeling burdened, which may result in depression, anxiety and other health problems. Many caregiver support interventions have been developed to ameliorate negative caregiver consequences with promising results. Early intervention and support for caregivers could prevent high levels of burden and psychological problems in the later stages of dementia. However, early-stage interventions may not be effective, and even do more harm than good if they do not fit the personal situation of the caregiver. Negative and stigmatizing information can hamper acceptance, while enhancing the positive, intact experiences may be effective in increasing caregiver self-efficacy. By increasing caregiver resilience through self-efficacy, an increase of psychological problems in a later stage may be prevented."

"A self-management approach focused on learning to positively manage life with dementia could facilitate the adaptation process to the new caregiving role in the early stages."

"With the growing gap between the number of people in need of support and available care professionals, eHealth interventions could serve as cost-effective alternatives for dementia caregiver support, with increased access and extended reach."

"Although e-health interventions for caregivers have been developed and evaluated, far most of them are aimed at dementia related problems in an advanced stage of the caregiver career and their overall quality of evidence is low. An iterative step-wise approach was employed to develop the blended care self-management internet-based program ‘Partner in Balance’ for early-stage dementia caregivers."

## 2a-ii) Scientific background, rationale: What is known about the (type of) system

Scientific background, rationale: What is known about the (type of) system that is the object of the study (be sure to discuss the use of similar systems for other conditions/diagnoses, if appropiate), motivation for the study, i.e. what are the reasons for and what is the context for this specific study, from which stakeholder viewpoint is the study performed, potential impact of findings [2]. Briefly justify the choice of the comparator.

| | 1 | 2 | 3 | 4 | 5 | |
|---|---|---|---|---|---|---|
| subitem not at all important | ○ | ○ | ○ | ○ | ◉ | essential |

## Does your paper address subitem 2a-ii? *

Copy and paste relevant sections from the manuscript (include quotes in quotation marks "like this" to indicate direct quotes from your manuscript), or elaborate on this item by providing additional information not in the ms, or briefly explain why the item is not applicable/relevant for your study

"Many caregiver support interventions have been developed to ameliorate negative caregiver consequences with promising results."

"Early intervention and support for caregivers could prevent high levels of burden and psychological problems in the later stages of dementia."

"By increasing caregiver resilience through self-efficacy, an increase of psychological problems in a later stage may be prevented. A self-management approach focused on learning to positively manage life with dementia could facilitate the adaptation process to the new caregiving role in the early stages."

"Blending face-to-face guidance with online support increases client-therapist connection and adherence. Although e-health interventions for caregivers have been developed and evaluated, far most of them are aimed at dementia related problems in an advanced stage of the caregiver career and their overall quality of evidence is low. An iterative step-wise approach was employed to develop the blended care self-management internet-based program ‘Partner in Balance’ for early-stage dementia caregivers."

## 2b) In INTRODUCTION: Specific objectives or hypotheses

## Does your paper address CONSORT subitem 2b? *

Copy and paste relevant sections from the manuscript (include quotes in quotation marks "like this" to indicate direct quotes from your manuscript), or elaborate on this item by providing additional information not in the ms, or briefly explain why the item is not applicable/relevant for your study

"The current study evaluated if ‘Partner in Balance’ is superior to a waiting-list control condition as evidenced by improved subjective self-confidence (self-efficacy and mastery), and lower levels of psychological complaints (symptoms of depression, anxiety and stress) post-intervention"

# METHODS

## 3a) Description of trial design (such as parallel, factorial) including allocation ratio

## Does your paper address CONSORT subitem 3a? *

Copy and paste relevant sections from the manuscript (include quotes in quotation marks "like this" to indicate direct quotes from your manuscript), or elaborate on this item by providing additional information not in the ms, or briefly explain why the item is not applicable/relevant for your study

This randomized controlled trial was carried out between 2014 and 2016 in the Netherlands. The “Partner in Balance” program was compared to a waiting-list control group receiving usual care. The Medical Ethics Committee of the Maastricht University Medical Center+ (MUMC+) approved this study (#12-4-059) and the study was registered in the Dutch trial register (NTR4748). The study protocol and supporting SPIRIT checklist are available.

"Following the baseline assessment, participants were randomly assigned to either “Partner in Balance” or the waiting-list control group receiving usual care, using a computerized random-number generator for block randomization with variable sizes of 4, 6 and 8. An independent research assistant who was blinded to the allocation of the treatment conducted the post-intervention assessments."

## 3b) Important changes to methods after trial commencement (such as eligibility criteria), with reasons

### Does your paper address CONSORT subitem 3b? *

Copy and paste relevant sections from the manuscript (include quotes in quotation marks "like this" to indicate direct quotes from your manuscript), or elaborate on this item by providing additional information not in the ms, or briefly explain why the item is not applicable/relevant for your study

Not applicable to the current study as the study followed the registered protocol.

### 3b-i) Bug fixes, Downtimes, Content Changes

Bug fixes, Downtimes, Content Changes: ehealth systems are often dynamic systems. A description of changes to methods therefore also includes important changes made on the intervention or comparator during the trial (e.g., major bug fixes or changes in the functionality or content) (5-iii) and other "unexpected events" that may have influenced study design such as staff changes, system failures/downtimes, etc. [2].

| | 1 | 2 | 3 | 4 | 5 | |
|---|---|---|---|---|---|---|
| subitem not at all important | ○ | ○ | ○ | ○ | ● | essential |

### Does your paper address subitem 3b-i?

Copy and paste relevant sections from the manuscript (include quotes in quotation marks "like this" to indicate direct quotes from your manuscript), or elaborate on this item by providing additional information not in the ms, or briefly explain why the item is not applicable/relevant for your study

Details on technological problems and content changes and its possible implications for intervention effectiveness are described in detail in the recently published process paper (JMIR MS #7666; PMID: 29258980)

## 4a) Eligibility criteria for participants

## Does your paper address CONSORT subitem 4a? *

Copy and paste relevant sections from the manuscript (include quotes in quotation marks "like this" to indicate direct quotes from your manuscript), or elaborate on this item by providing additional information not in the ms, or briefly explain why the item is not applicable/relevant for your study

"Family caregivers of people with (very) mild dementia of all subtypes (Clinical Dementia Rating (CDR) score 0.5-1).."

"Caregivers were included if they had access to the Internet at home, basic skills in the use of computers, and provided written informed consent. Potential participants with insufficient cognitive abilities to engage in the online self-management program; overburdened or with severe health problems as determined by study staff; or who cared for people with dementia caused by human immunodeficiency virus (HIV), acquired brain impairment, Down syndrome, chorea associated with Huntington's disease or alcohol abuse were excluded from participation. In- and exclusion was based on clinical judgment of the referrer, based on his/her experience with the target group. Both spouses and other caregivers could be included, e.g., children, as long as they met the criteria above and were >18 years. Details on the recruitment procedure are described elsewhere."

## 4a-i) Computer / Internet literacy

Computer / Internet literacy is often an implicit "de facto" eligibility criterion - this should be explicitly clarified.

| | 1 | 2 | 3 | 4 | 5 | |
|---|---|---|---|---|---|---|
| subitem not at all important | ○ | ○ | ○ | ○ | ◉ | essential |

## Does your paper address subitem 4a-i?

Copy and paste relevant sections from the manuscript (include quotes in quotation marks "like this" to indicate direct quotes from your manuscript), or elaborate on this item by providing additional information not in the ms, or briefly explain why the item is not applicable/relevant for your study

"Caregivers were included if they had access to the Internet at home, basic skills in the use of computers.."

## 4a-ii) Open vs. closed, web-based vs. face-to-face assessments:

Open vs. closed, web-based vs. face-to-face assessments: Mention how participants were recruited (online vs. offline), e.g., from an open access website or from a clinic, and clarify if this was a purely web-based trial, or there were face-to-face components (as part of the intervention or for assessment), i.e., to what degree got the study team to know the participant. In online-only trials, clarify if participants were quasi-anonymous and whether having multiple identities was possible or whether technical or logistical measures (e.g., cookies, email confirmation, phone calls) were used to detect/prevent these.

| | 1 | 2 | 3 | 4 | 5 | |
|---|---|---|---|---|---|---|
| subitem not at all important | ○ | ○ | ○ | ○ | ◉ | essential |

## Does your paper address subitem 4a-ii? *

Copy and paste relevant sections from the manuscript (include quotes in quotation marks "like this" to indicate direct quotes from your manuscript), or elaborate on this item by providing additional information not in the ms, or briefly explain why the item is not applicable/relevant for your study

"Family caregivers of people with (very) mild dementia of all subtypes (Clinical Dementia Rating (CDR) score 0.5-1) were recruited from memory clinics (MUMC+, Elkerliek Hospital Helmond, Catharina Hospital Eindhoven) and ambulatory mental health clinics (Virenze-RIAGG Maastricht, MET ggz Roermond) in the south of the Netherlands, via caregiver support services, and via the website of the Dutch Alzheimer Association (www.alzheimernederland.nl)."

"For this study data from the baseline visit (T0) and after 8 weeks (T1) were compared. These data were collected in writing by an independent research assistant who was unknown to the allocation of the treatment, separately from the coach visits."

## 4a-iii) Information giving during recruitment

Information given during recruitment. Specify how participants were briefed for recruitment and in the informed consent procedures (e.g., publish the informed consent documentation as appendix, see also item X26), as this information may have an effect on user self-selection, user expectation and may also bias results.

| | 1 | 2 | 3 | 4 | 5 | |
|---|---|---|---|---|---|---|
| subitem not at all important | ○ | ○ | ○ | ○ | ◉ | essential |

## Does your paper address subitem 4a-iii?

Copy and paste relevant sections from the manuscript (include quotes in quotation marks "like this" to indicate direct quotes from your manuscript), or elaborate on this item by providing additional information not in the ms, or briefly explain why the item is not applicable/relevant for your study

"The Medical Ethics Committee of the Maastricht University Medical Center+ (MUMC+) approved this study (#12-4-059) and the study was registered in the Dutch trial register (NTR4748). The study protocol including informed consent procedure and supporting SPIRIT checklist are available" (PMID: 27142676)

## 4b) Settings and locations where the data were collected

## Does your paper address CONSORT subitem 4b? *

Copy and paste relevant sections from the manuscript (include quotes in quotation marks "like this" to indicate direct quotes from your manuscript), or elaborate on this item by providing additional information not in the ms, or briefly explain why the item is not applicable/relevant for your study

"These data were collected in writing by an independent research assistant who was unknown to the allocation of the treatment, separately from the coach visits."

Details on data collection setting and location are described in detail in the recently published process paper (JMIR MS #7666; PMID: 29258980)

## 4b-i) Report if outcomes were (self-)assessed through online questionnaires

Clearly report if outcomes were (self-)assessed through online questionnaires (as common in web-based trials) or otherwise.

| | 1 | 2 | 3 | 4 | 5 | |
|---|---|---|---|---|---|---|
| subitem not at all important | ○ | ○ | ○ | ○ | ◉ | essential |

## Does your paper address subitem 4b-i? *

Copy and paste relevant sections from the manuscript (include quotes in quotation marks "like this" to indicate direct quotes from your manuscript), or elaborate on this item by providing additional information not in the ms, or briefly explain why the item is not applicable/relevant for your study

"For this study self-report data from the baseline visit (T0) and after 8 weeks (T1) were compared. These data were collected in writing by an independent research assistant who was unknown to the allocation of the treatment, separately from the coach visits."

## 4b-ii) Report how institutional affiliations are displayed

Report how institutional affiliations are displayed to potential participants [on ehealth media], as affiliations with prestigious hospitals or universities may affect volunteer rates, use, and reactions with regards to an intervention.(Not a required item – describe only if this may bias results)

| | 1 | 2 | 3 | 4 | 5 | |
|---|---|---|---|---|---|---|
| subitem not at all important | ○ | ○ | ○ | ◉ | ○ | essential |

## Does your paper address subitem 4b-ii?

Copy and paste relevant sections from the manuscript (include quotes in quotation marks "like this" to indicate direct quotes from your manuscript), or elaborate on this item by providing additional information not in the ms, or briefly explain why the item is not applicable/relevant for your study

Details on recruitment and intervention description are described in detail in the recently published process paper (JMIR MS #7666; PMID: 29258980)

## 5) The interventions for each group with sufficient details to allow replication, including how and when they were actually administered

### 5-i) Mention names, credential, affiliations of the developers, sponsors, and owners

Mention names, credential, affiliations of the developers, sponsors, and owners [6] (if authors/evaluators are owners or developer of the software, this needs to be declared in a “Conflict of interest” section or mentioned elsewhere in the manuscript).

| | 1 | 2 | 3 | 4 | 5 | |
|---|---|---|---|---|---|---|
| subitem not at all important | ○ | ○ | ○ | ○ | ○ | essential |

## Does your paper address subitem 5-i?

Copy and paste relevant sections from the manuscript (include quotes in quotation marks "like this" to indicate direct quotes from your manuscript), or elaborate on this item by providing additional information not in the ms, or briefly explain why the item is not applicable/relevant for your study

"Experimental group: Partner in Balance (PiB). In short, the blended care self-management program PiB consists of: (1) a face-to-face intake session with a personal coach to familiarize participants with the program, set goals, and select preferred module themes; (2) tailored online thematic modules, including psychoeducation, behavioral modeling, reflective assignments, change plans, and email feedback from the coach over 8 weeks; and (3) a face-to-face evaluation session with the coach evaluating previously set goals. Module themes are aceptance, balance in activities, communication with family member and environment, coping with stress, focusing on the positive, insecurities and rumination, self-understanding, the changing family member, and social relations and support. Figure 1 shows a screenshot of the module themes in the program. The participants choose 4 modules; 2 weeks were allocated for each module. However, the participants were allowed to complete the modules at their own pace in accordance with the self-management approach.22 The personal page and modules remained accessible for participants after the intervention period. The personal coaches were trained, experienced professionals (psychologists and psychiatric nurses) from one of the participating organizations. They attented a 2-hour training in self-management techniques, goal setting and online help, and regular supervision meetings. Their tasks were familiarizing participants with the online program, supporting them in module choice and goal setting, and providing feedback on the self-reflective assignments through the online messaging portal in the program."

"Control group: waiting-list. The waiting-list group received usual care consisting of non-frequent counseling during 8 weeks. They received the same pre-test and post-test attention from the research team as the experimental group. After they completed the post-test assessment, they were given the opportunity to follow PiB."

## 5-ii) Describe the history/development process

Describe the history/development process of the application and previous formative evaluations (e.g., focus groups, usability testing), as these will have an impact on adoption/use rates and help with interpreting results.

| | 1 | 2 | 3 | 4 | 5 | |
|---|---|---|---|---|---|---|
| subitem not at all important | ○ | ○ | ○ | ○ | ◉ | essential |

## Does your paper address subitem 5-ii?

Copy and paste relevant sections from the manuscript (include quotes in quotation marks "like this" to indicate direct quotes from your manuscript), or elaborate on this item by providing additional information not in the ms, or briefly explain why the item is not applicable/relevant for your study

"Detailed information about the program components and development is presented elsewhere": In the article published in JMIR Res Protoc (PMID: 26932438) we described the development of "Partner in Balance" following the iterative process of the new Medical Research Counsel (MRC) Framework

## 5-iii) Revisions and updating

Revisions and updating. Clearly mention the date and/or version number of the application/intervention (and comparator, if applicable) evaluated, or describe whether the intervention underwent major changes during the evaluation process, or whether the development and/or content was "frozen" during the trial. Describe dynamic components such as news feeds or changing content which may have an impact on the replicability of the intervention (for unexpected events see item 3b).

| | 1 | 2 | 3 | 4 | 5 | |
|---|---|---|---|---|---|---|
| subitem not at all important | ○ | ○ | ○ | ○ | ◉ | essential |

## Does your paper address subitem 5-iii?

Copy and paste relevant sections from the manuscript (include quotes in quotation marks "like this" to indicate direct quotes from your manuscript), or elaborate on this item by providing additional information not in the ms, or briefly explain why the item is not applicable/relevant for your study

In the article published in JMIR Res Protoc (PMID: 26932438) we described the development of "Partner in Balance" following the iterative process of the new Medical Research Counsel (MRC) Framework

Details on intervention and trial process are described in detail in the recently published process paper (JMIR MS #7666; PMID: 29258980)

## 5-iv) Quality assurance methods

Provide information on quality assurance methods to ensure accuracy and quality of information provided [1], if applicable.

| | 1 | 2 | 3 | 4 | 5 | |
|---|---|---|---|---|---|---|
| subitem not at all important | ○ | ○ | ○ | ○ | ◉ | essential |

## Does your paper address subitem 5-iv?

Copy and paste relevant sections from the manuscript (include quotes in quotation marks "like this" to indicate direct quotes from your manuscript), or elaborate on this item by providing additional information not in the ms, or briefly explain why the item is not applicable/relevant for your study

"The Medical Ethics Committee of the Maastricht University Medical Center+ (MUMC+) approved this study (#12-4-059) and the study was registered in the Dutch trial register (NTR4748). The study protocol and supporting SPIRIT checklist are available."

"Following the baseline assessment, participants were randomly assigned to either “Partner in Balance” or the waiting-list control group receiving usual care, using a computerized random-number generator for block randomization with variable sizes of 4, 6 and 8. An independent research assistant who was blinded to the allocation of the treatment conducted the post-intervention assessments."

## 5-v) Ensure replicability by publishing the source code, and/or providing screenshots/screen-capture video, and/or providing flowcharts of the algorithms used

Ensure replicability by publishing the source code, and/or providing screenshots/screen-capture video, and/or providing flowcharts of the algorithms used. Replicability (i.e., other researchers should in principle be able to replicate the study) is a hallmark of scientific reporting.

| | 1 | 2 | 3 | 4 | 5 | |
|---|---|---|---|---|---|---|
| subitem not at all important | ○ | ○ | ○ | ○ | ◉ | essential |

## Does your paper address subitem 5-v?

Copy and paste relevant sections from the manuscript (include quotes in quotation marks "like this" to indicate direct quotes from your manuscript), or elaborate on this item by providing additional information not in the ms, or briefly explain why the item is not applicable/relevant for your study

"Experimental group: Partner in Balance (PiB). Detailed information about the program components and development is presented elsewhere.21 In short, the blended care self-management program PiB (www.partnerinbalans.nl) consists of: (1) a face-to-face intake session with a personal coach to familiarize participants with the program, set goals, and select preferred module themes; (2) tailored online thematic modules, including psychoeducation, behavioral modeling, reflective assignments, change plans, and email feedback from the coach over 8 weeks; and (3) a face-to-face evaluation session with the coach evaluating previously set goals. Module themes are aceptance, balance in activities, communication with family member and environment, coping with stress, focusing on the positive, insecurities and rumination, self-understanding, the changing family member, and social relations and support. Figure 1 shows a screenshot of the module themes in the program. The participants choose 4 modules; 2 weeks were allocated for each module. However, the participants were allowed to complete the modules at their own pace in accordance with the self-management approach.22 The personal page and modules remained accessible for participants after the intervention period. The personal coaches were trained, experienced professionals (psychologists and psychiatric nurses) from one of the participating organizations. They attented a 2-hour training in self-management techniques, goal setting and online help, and regular supervision meetings. Their tasks were familiarizing participants with the online program, supporting them in module choice and goal setting, and providing feedback on the self-reflective assignments through the online messaging portal in the program (see Figure 2)."

## 5-vi) Digital preservation

Digital preservation: Provide the URL of the application, but as the intervention is likely to change or disappear over the course of the years; also make sure the intervention is archived (Internet Archive, webcitation.org, and/or publishing the source code or screenshots/videos alongside the article). As pages behind login screens cannot be archived, consider creating demo pages which are accessible without login.

| | 1 | 2 | 3 | 4 | 5 | |
|---|---|---|---|---|---|---|
| subitem not at all important | ○ | ○ | ○ | ○ | ◉ | essential |

## Does your paper address subitem 5-vi?

Copy and paste relevant sections from the manuscript (include quotes in quotation marks "like this" to indicate direct quotes from your manuscript), or elaborate on this item by providing additional information not in the ms, or briefly explain why the item is not applicable/relevant for your study

Experimental group: Partner in Balance (PiB). Detailed information about the program components and development is presented elsewhere.21 In short, the blended care self-management program PiB (www.partnerinbalans.nl, archived at http://www.webcitation.org/6vu442qdc) consists of: (1) a face-to-face intake session with a personal coach to familiarize participants with the program, set goals, and select preferred module themes; (2) tailored online thematic modules, including psychoeducation, behavioral modeling, reflective assignments, change plans, and email feedback from the coach over 8 weeks; and (3) a face-to-face evaluation session with the coach evaluating previously set goals. Module themes are aceptance, balance in activities, communication with family member and environment, coping with stress, focusing on the positive, insecurities and rumination, self-understanding, the changing family member, and social relations and support. Figure 1 shows a screenshot of the module themes in the program. The participants choose 4 modules; 2 weeks were allocated for each module. However, the participants were allowed to complete the modules at their own pace in accordance with the self-management approach.22 The personal page and modules remained accessible for participants after the intervention period. The personal coaches were trained, experienced professionals (psychologists and psychiatric nurses) from one of the participating organizations. They attented a 2-hour training in self-management techniques, goal setting and online help, and regular supervision meetings. Their tasks were familiarizing participants with the online program, supporting them in module choice and goal setting, and providing feedback on the self-reflective assignments through the online messaging portal in the program (see Figure 2).

## 5-vii) Access

Access: Describe how participants accessed the application, in what setting/context, if they had to pay (or were paid) or not, whether they had to be a member of specific group. If known, describe how participants obtained "access to the platform and Internet" [1]. To ensure access for editors/reviewers/readers, consider to provide a "backdoor" login account or demo mode for reviewers/readers to explore the application (also important for archiving purposes, see vi).

| | 1 | 2 | 3 | 4 | 5 | |
|---|---|---|---|---|---|---|
| subitem not at all important | ○ | ○ | ○ | ○ | ○ | essential |

## Does your paper address subitem 5-vii? *

Copy and paste relevant sections from the manuscript (include quotes in quotation marks "like this" to indicate direct quotes from your manuscript), or elaborate on this item by providing additional information not in the ms, or briefly explain why the item is not applicable/relevant for your study

Details on the recruitment process are described in detail in the recently published process paper (JMIR MS #7666; PMID: 29258980)

## 5-viii) Mode of delivery, features/functionalities/components of the intervention and comparator, and the theoretical framework

Describe mode of delivery, features/functionalities/components of the intervention and comparator, and the theoretical framework [6] used to design them (instructional strategy [1], behaviour change techniques, persuasive features, etc., see e.g., [7, 8] for terminology). This includes an in-depth description of the content (including where it is coming from and who developed it) [1]," whether [and how] it is tailored to individual circumstances and allows users to track their progress and receive feedback" [6]. This also includes a description of communication delivery channels and – if computer-mediated communication is a component – whether communication was synchronous or asynchronous [6]. It also includes information on presentation strategies [1], including page design principles, average amount of text on pages, presence of hyperlinks to other resources, etc. [1].

| | 1 | 2 | 3 | 4 | 5 | |
|---|---|---|---|---|---|---|
| subitem not at all important | ○ | ○ | ○ | ○ | ◉ | essential |

## Does your paper address subitem 5-viii? *

Copy and paste relevant sections from the manuscript (include quotes in quotation marks "like this" to indicate direct quotes from your manuscript), or elaborate on this item by providing additional information not in the ms, or briefly explain why the item is not applicable/relevant for your study

This item was addressed in detail in the intervention design paper published in [illegible]

JMIR Res Prot (JMIR Res Prot MS #5142; PMID: PMC4795319)

"Methods
Intervention content was proposed by authors LMMB and MEdV based on a literature review [10], EDC needs [24], identified themes in Step 1, and conceptual frameworks on self-management. The Stress and Coping paradigm [25] served as the theoretical basis for the content of the modules. According to this model, stress is experienced when a person perceives that the demands (caring for a person with dementia) exceed their personal and social resources. Caregivers' responses to their stress situation might be mediated by their understanding of the situation and their beliefs about their ability to cope. The latter fits Bandura's [26] concept of self-efficacy (belief in one's capabilities). Consistent with this theory, models of dementia management emphasize the need to maintain self-worth and control [28]. An intervention aimed at increasing self-efficacy should not only educate the caregiver, but should also foster self-management by combining education with problem-solving skills, and work toward a change in behavior [36].
Results
The proposed self-management intervention program "Partner in Balance" (PiB) encourages caregivers to actively manage their lives and identify solutions for their specific needs [37]. Increasing knowledge, identifying and setting goals, and learning skills to achieve these previously set goals served as the basis for the intervention program. Module content was focused on role management (eg, balancing activities in daily life) and emotional management (eg, dealing with fear and insecurity about the future) [38]. Formulating, planning, and executing personal goals can be learned using a proactive 5-step change plan (Textbox 1) often used in self-management [38], which was integrated into each module. By formulating and planning a personal change plan, caregivers learn to anticipate on stressful situations and gain confidence in their ability to take care of the situation and themselves [38]. Because caregivers greatly varied in their needs, personal goals, and interest, a flexible choice of modules was used. Successful elements that were identified in the literature review [10], including tailored caregiving strategies

the literature review [illegible], including tailored caregiving strategies
and contact with a coach and/or other caregivers, were included
in the program content likewise."

## 5-ix) Describe use parameters

Describe use parameters (e.g., intended “doses” and optimal timing for use). Clarify what instructions or recommendations were given to the user, e.g., regarding timing, frequency, heaviness of use, if any, or was the intervention used ad libitum.

| | 1 | 2 | 3 | 4 | 5 | |
|---|---|---|---|---|---|---|
| subitem not at all important | ○ | ○ | ○ | ○ | ◉ | essential |

## Does your paper address subitem 5-ix?

Copy and paste relevant sections from the manuscript (include quotes in quotation marks "like this" to indicate direct quotes from your manuscript), or elaborate on this item by providing additional information not in the ms, or briefly explain why the item is not applicable/relevant for your study

Details on the intended dose and instructions for participants are described in the recently published process paper (JMIR MS #7666; PMID: 29258980)

"In short, the blended care self-management program PiB consists of the following: (1) a face-to-face intake session with a personal coach to familiarize participants with the program, set goals with the goal attainment scaling (GAS) method, and select preferred module themes [26]; (2) tailored online thematic modules, including psychoeducation, behavioral modeling, reflective assignments, change plans, and email feedback from the coach over 8 weeks; and (3) a face-to-face evaluation session with the coach evaluating previously set goals."

"The participants choose 4 modules; 2
weeks were allocated for each module. However, the participants were allowed to complete the modules at their own pace in accordance with the self-management approach [27]. The personal page and modules remained accessible for participants after the intervention period. The control group consisted of an 8-week waiting list while receiving usual care (nonfrequent counseling). After the posttest assessment, they were given the opportunity to follow PiB."

opportunity to follow PIB.

"Regarding the dose delivered, out of the program completers (n=49), 87.8% (43/49) completed all 4 modules, 6.1% (3/49) completed 3 modules, and 6.1% (3/49) completed 2 modules. The tracked usage data showed 21,946 clicks per module, including exploring the website (2444 clicks), viewing the psychological educative information (3922 clicks), completing the assignments and change plan (8748 clicks), contacting the personal coach (6489 clicks), and visiting the discussion forum (310 clicks). The total intervention time ranged from 4 to 32 weeks (mean 13.9 [SD 6.8]). Reasons for intervention period variability were holidays, illness, busy schedules, and technical difficulties. Following the intervention period, 77.6% of the program completers (38/49) requested access to the additional modules with (16/49) or without (33/49) the coach at their disposal for questions."

## 5-x) Clarify the level of human involvement

Clarify the level of human involvement (care providers or health professionals, also technical assistance) in the e-intervention or as co-intervention (detail number and expertise of professionals involved, if any, as well as “type of assistance offered, the timing and frequency of the support, how it is initiated, and the medium by which the assistance is delivered”. It may be necessary to distinguish between the level of human involvement required for the trial, and the level of human involvement required for a routine application outside of a RCT setting (discuss under item 21 – generalizability).

| | 1 | 2 | 3 | 4 | 5 | |
|---|---|---|---|---|---|---|
| subitem not at all important | ○ | ○ | ○ | ○ | ◉ | essential |

## Does your paper address subitem 5-x?

Copy and paste relevant sections from the manuscript (include quotes in quotation marks "like this" to indicate direct quotes from your manuscript), or elaborate on this item by providing additional information not in the ms, or briefly explain why the item is not applicable/relevant for your study

"Detailed information about the program components and development is presented elsewhere.21 In short, the blended care self-management program PiB (www.partnerinbalans.nl, archived at http://www.webcitation.org/6vu442qdc) consists of: (1) a face-to-face intake session with a personal coach to familiarize participants with the program, set goals, and select preferred module themes; (2) tailored online thematic modules, including psychoeducation, behavioral modeling, reflective assignments, change plans, and email feedback from the coach over 8 weeks; and (3) a face-to-face evaluation session with the coach evaluating previously set goals."

"The personal coaches were trained, experienced professionals (psychologists and psychiatric nurses) from one of the participating organizations. They attented a 2-hour training in self-management techniques, goal setting and online help, and regular supervision meetings. Their tasks were familiarizing participants with the online program, supporting them in module choice and goal setting, and providing feedback on the self-reflective assignments through the online messaging portal in the program"

"For this study self-report data from the baseline visit (T0) and after 8 weeks (T1) were compared. These data were collected in writing by an independent research assistant who was unknown to the allocation of the treatment, separately from the coach visits."

## 5-xi) Report any prompts/reminders used

Report any prompts/reminders used: Clarify if there were prompts (letters, emails, phone calls, SMS) to use the application, what triggered them, frequency etc. It may be necessary to distinguish between the level of prompts/reminders required for the trial, and the level of prompts/reminders for a routine application outside of a RCT setting (discuss under item 21 – generalizability).

| | 1 | 2 | 3 | 4 | 5 | |
|---|---|---|---|---|---|---|
| subitem not at all important | ○ | ○ | ○ | ◉ | ○ | essential |

## Does your paper address subitem 5-xi? *

Copy and paste relevant sections from the manuscript (include quotes in quotation marks "like this" to indicate direct quotes from your manuscript), or elaborate on this item by providing additional information not in the ms, or briefly explain why the item is not applicable/relevant for your study

This item was described in the recently published process paper (JMIR MS #7666; PMID: 29258980)

"The participants choose 4 modules; 2
weeks were allocated for each module. However, the participants were allowed to complete the modules at their own pace in accordance with the self-management approach [27]. The personal page and modules remained accessible for participants after the intervention period. The control group consisted of an 8-week waiting list while receiving usual care (nonfrequent counseling). After the posttest assessment, they were given the opportunity to follow PiB."

## 5-xii) Describe any co-interventions (incl. training/support)

Describe any co-interventions (incl. training/support): Clearly state any interventions that are provided in addition to the targeted eHealth intervention, as ehealth intervention may not be designed as stand-alone intervention. This includes training sessions and support [1]. It may be necessary to distinguish between the level of training required for the trial, and the level of training for a routine application outside of a RCT setting (discuss under item 21 – generalizability.

| | 1 | 2 | 3 | 4 | 5 | |
|---|---|---|---|---|---|---|
| subitem not at all important | ○ | ○ | ○ | ○ | ● | essential |

## Does your paper address subitem 5-xii? *

Copy and paste relevant sections from the manuscript (include quotes in quotation marks "like this" to indicate direct quotes from your manuscript), or elaborate on this item by providing additional information not in the ms, or briefly explain why the item is not applicable/relevant for your study

"Control group: waiting-list. The waiting-list group received usual care consisting of non-frequent counseling during 8 weeks. They received the same pre-test and post-test attention from the research team as the experimental group. After they completed the post-test assessment, they were given the opportunity to follow PiB."

## 6a) Completely defined pre-specified primary and secondary outcome measures, including how and when they were assessed

### Does your paper address CONSORT subitem 6a? *

Copy and paste relevant sections from the manuscript (include quotes in quotation marks "like this" to indicate direct quotes from your manuscript), or elaborate on this item by providing additional information not in the ms, or briefly explain why the item is not applicable/relevant for your study

"For this study self-report data from the baseline visit (T0) and after 8 weeks (T1) were compared. These data were collected in writing by an independent research assistant who was unknown to the allocation of the treatment, separately from the coach visits.
The primary proximal outcome was caregiver self-efficacy and primary distal outcome was depressive symptoms. Caregiver self-efficacy was measured with The Caregiver Self-efficacy Scale (CSES),23 measuring care management self-efficacy (4 items) and service use self-efficacy (5 items). Care management self-efficacy scores theoretically range from 4-40 and service use self-efficacy from 5-50. Higher scores on the CSES indicate higher levels of self-efficacy. The 20-item Centre for Epidemiological Studies Depression Scale (CES-D)24 was used to measure depressive symptoms. Total scores range from 0-60; higher scores indicate more symptoms. Secondary outcomes were mastery, psychological complaints (anxiety and perceived stress), and quality of life. Mastery was measured with the 7-item Pearlin Mastery Scale (PMS).26 The total score ranges from 7-35; higher scores indicate higher levels of mastery. The 7-item Hospital and Anxiety Depression Scale anxiety subscale (HADS-A)27 rates symptoms of anxiety. Scores theoretically range from 0-21 with higher scores indicating more symptoms. Quality of life was measured on five attributes with the Investigating Choice Experiments for the Preferences of Older People CAPability measure for Older people (ICECAP-O).28 The value system for the 1024 states uses a best-worst scaling valuation method, providing a single summary score, anchored at zero ('no capability') and 1.0 ('full capability')."

## 6a-i) Online questionnaires: describe if they were validated for online use and apply CHERRIES items to describe how the questionnaires were designed/deployed

If outcomes were obtained through online questionnaires, describe if they were validated for online use and apply CHERRIES items to describe how the questionnaires were designed/deployed [9].

| | 1 | 2 | 3 | 4 | 5 | |
|---|---|---|---|---|---|---|
| subitem not at all important | ○ | ◉ | ○ | ○ | ○ | essential |

## Does your paper address subitem 6a-i?

Copy and paste relevant sections from manuscript text

Not applicable for our study, we used offline questionnaires.

## 6a-ii) Describe whether and how "use" (including intensity of use/dosage) was defined/measured/monitored

Describe whether and how "use" (including intensity of use/dosage) was defined/measured/monitored (logins, logfile analysis, etc.). Use/adoption metrics are important process outcomes that should be reported in any ehealth trial.

| | 1 | 2 | 3 | 4 | 5 | |
|---|---|---|---|---|---|---|
| subitem not at all important | ○ | ○ | ○ | ○ | ◉ | essential |

## Does your paper address subitem 6a-ii?

Copy and paste relevant sections from manuscript text

This item was described in the recently published process paper (JMIR MS #7666; PMID: 29258980)

"Furthermore, usage of the
website (clickstream per intervention component) was tracked.
Clickstream data are information trails that users leave behind
while visiting the website. As participants clicked anywhere on
the webpage, this action was captured in a log file. Clicks
represent the number of times a page has been viewed and can
be used to track which elements of the website were visited

most often [29]."

"Performance According to the Protocol
Intervention performance according to protocol comprised a face-to-face intake session, online modules over 8 weeks, individualized feedback via email for each module, and a face-to-face evaluation session. A total of 10 out of 13 coaches reported performance according to the protocol (77%), and 3 out of 13 reported deviations in intervention time, structure, and feedback (23%). Intervention time was reported to be longer (n=2) or shorter (n=1) than 8 weeks, and the module structure was consumed differently than intended (n=2) or feedback was given by telephone (n=2) or in person (n=1). Reasons to deviate from the protocol included caregiver pace and understanding of the program structure (n=3), illness (n=1), holiday leave (n=1), changes in work load and hours (n=1), personal family emergencies (n=1), and struggling to verbalize feedback in an email (n=1).
Regarding the dose delivered, out of the program completers (n=49), 87.8% (43/49) completed all 4 modules, 6.1% (3/49) completed 3 modules, and 6.1% (3/49) completed 2 modules. The tracked usage data showed 21,946 clicks per module, including exploring the website (2444 clicks), viewing the psychological educative information (3922 clicks), completing the assignments and change plan (8748 clicks), contacting the personal coach (6489 clicks), and visiting the discussion forum (310 clicks). The total intervention time ranged from 4 to 32 weeks (mean 13.9 [SD 6.8]). Reasons for intervention period variability were holidays, illness, busy schedules, and technical difficulties. Following the intervention period, 77.6% of the program completers (38/49) requested access to the additional modules with (16/49) or without (33/49) the coach at their disposal for questions."

## 6a-iii) Describe whether, how, and when qualitative feedback from participants was obtained

Describe whether, how, and when qualitative feedback from participants was obtained (e.g., through emails, feedback forms, interviews, focus groups).

| | 1 | 2 | 3 | 4 | 5 | |
|---|---|---|---|---|---|---|
| subitem not at all important | ○ | ○ | ○ | ○ | ◉ | essential |

## Does your paper address subitem 6a-iii?

Copy and paste relevant sections from manuscript text

This item was described in the recently published process paper (JMIR MS #7666; PMID: 29258980)

"Data from the perspective of the participants were collected postintervention with a semistructured interview (Textbox 1) with participants in both the intervention and waiting-list group. The interviews were audiotaped with the participants' permission"

## 6b) Any changes to trial outcomes after the trial commenced, with reasons

## Does your paper address CONSORT subitem 6b? *

Copy and paste relevant sections from the manuscript (include quotes in quotation marks "like this" to indicate direct quotes from your manuscript), or elaborate on this item by providing additional information not in the ms, or briefly explain why the item is not applicable/relevant for your study

Not applicable for this study, the trial outcomes were not changed.

## 7a) How sample size was determined

NPT: When applicable, details of whether and how the clustering by care provides or centers was addressed

## 7a-i) Describe whether and how expected attrition was taken into account when calculating the sample size

Describe whether and how expected attrition was taken into account when calculating the sample size.

| | 1 | 2 | 3 | 4 | 5 | |
|---|---|---|---|---|---|---|
| subitem not at all important | ○ | ○ | ○ | ○ | ◉ | essential |

## Does your paper address subitem 7a-i?

Copy and paste relevant sections from manuscript title (include quotes in quotation marks "like this" to indicate direct quotes from your manuscript), or elaborate on this item by providing additional information not in the ms, or briefly explain why the item is not applicable/relevant for your study

"We aimed to enroll 80 participants (40 participants per group), based on previous online intervention studies in caregivers of people with dementia with the Caregiver Self-efficacy Scale (CSES) as outcome measure, on the basis of repeated measures, within-between interaction with a mean effect size of 0.2 [55], assuming alpha 0.05, power 85% and 25% loss to follow-up."

## 7b) When applicable, explanation of any interim analyses and stopping guidelines

## Does your paper address CONSORT subitem 7b? *

Copy and paste relevant sections from the manuscript (include quotes in quotation marks "like this" to indicate direct quotes from your manuscript), or elaborate on this item by providing additional information not in the ms, or briefly explain why the item is not applicable/relevant for your study

Not applicable for this study, data were analysed after the trial was completed.

## 8a) Method used to generate the random allocation sequence

NPT: When applicable, how care providers were allocated to each trial group

## Does your paper address CONSORT subitem 8a? *

Copy and paste relevant sections from the manuscript (include quotes in quotation marks "like this" to indicate direct quotes from your manuscript), or elaborate on this item by providing additional information not in the ms, or briefly explain why the item is not applicable/relevant for your study

"Following the baseline assessment, participants were randomly assigned to either “Partner in Balance” or the waiting-list control group receiving usual care by the first author, using a computerized random-number generator for block randomization with variable sizes of 4, 6 and 8. An independent research assistant who was blinded to the allocation of the treatment conducted the post-intervention assessments."

## 8b) Type of randomisation; details of any restriction (such as blocking and block size)

## Does your paper address CONSORT subitem 8b? *

Copy and paste relevant sections from the manuscript (include quotes in quotation marks "like this" to indicate direct quotes from your manuscript), or elaborate on this item by providing additional information not in the ms, or briefly explain why the item is not applicable/relevant for your study

"Following the baseline assessment, participants were randomly assigned to either “Partner in Balance” or the waiting-list control group receiving usual care by the first author, using a computerized random-number generator for block randomization with variable sizes of 4, 6 and 8. An independent research assistant who was blinded to the allocation of the treatment conducted the post-intervention assessments."

## 9) Mechanism used to implement the random allocation sequence (such as sequentially numbered containers), describing any steps taken to conceal the sequence until interventions were assigned

## Does your paper address CONSORT subitem 9? *

Copy and paste relevant sections from the manuscript (include quotes in quotation marks "like this" to indicate direct quotes from your manuscript), or elaborate on this item by providing additional information not in the ms, or briefly explain why the item is not applicable/relevant for your study

"An independent research assistant who was blinded to the allocation of the treatment conducted the post-intervention assessments."

## 10) Who generated the random allocation sequence, who enrolled participants, and who assigned participants to interventions

## Does your paper address CONSORT subitem 10? *

Copy and paste relevant sections from the manuscript (include quotes in quotation marks "like this" to indicate direct quotes from your manuscript), or elaborate on this item by providing additional information not in the ms, or briefly explain why the item is not applicable/relevant for your study

"Following the baseline assessment, participants were randomly assigned to either “Partner in Balance” or the waiting-list control group receiving usual care by the first author, using a computerized random-number generator for block randomization with variable sizes of 4, 6 and 8. An independent research assistant who was blinded to the allocation of the treatment conducted the post-intervention assessments."

## 11a) If done, who was blinded after assignment to interventions (for example, participants, care providers, those assessing outcomes) and how

NPT: Whether or not administering co-interventions were blinded to group assignment

## 11a-i) Specify who was blinded, and who wasn't

Specify who was blinded, and who wasn't. Usually, in web-based trials it is not possible to blind the participants [1, 3] (this should be clearly acknowledged), but it may be possible to blind outcome assessors, those doing data analysis or those administering co-interventions (if any).

| | 1 | 2 | 3 | 4 | 5 | |
|---|---|---|---|---|---|---|
| subitem not at all important | ○ | ○ | ○ | ○ | ◉ | essential |

## Does your paper address subitem 11a-i? *

Copy and paste relevant sections from the manuscript (include quotes in quotation marks "like this" to indicate direct quotes from your manuscript), or elaborate on this item by providing additional information not in the ms, or briefly explain why the item is not applicable/relevant for your study

This item was described in the recently published process paper (JMIR MS #7666; PMID: 29258980)

"Following the baseline assessment, the participants were randomly allocated to either PiB or the waiting-list control group. The researcher (LMMB) not involved in the assessments performed the allocation. A research assistant blind to the allocation conducted the assessments and recorded the blinding success and reason for the possible unmasking on the case record form. At T1, 68 participants had completed the postintervention or postwaiting list assessment and blinding was intact for 46% (31/68), unsuccessful for 49% (33/68), and for 7% (5/68), a conjecture of allocation was expressed."

"It was not possible to blind the participants because of obvious differences between the interventions in content and mode of delivery."

## 11a-ii) Discuss e.g., whether participants knew which intervention was the “intervention of interest” and which one was the “comparator”

Informed consent procedures (4a-ii) can create biases and certain expectations - discuss e.g., whether participants knew which intervention was the “intervention of interest” and which one was the “comparator”.

| | 1 | 2 | 3 | 4 | 5 | |
|---|---|---|---|---|---|---|
| subitem not at all important | ○ | ○ | ○ | ○ | ◉ | essential |

## Does your paper address subitem 11a-ii?

Copy and paste relevant sections from the manuscript (include quotes in quotation marks "like this" to indicate direct quotes from your manuscript), or elaborate on this item by providing additional information not in the ms, or briefly explain why the item is not applicable/relevant for your study

This item was described in the recently published process paper (JMIR MS #7666; PMID: 29258980)

"A total of 163 caregivers were invited to participate.
See Figure 1 for the study flowchart. If they expressed interest,
family caregivers (n=138) received a detailed information letter.
Of the information recipients, IC was signed by 58.0% (80/138).
Of the 163 recruited caregivers, 154 were eligible for
participation. The participation rate of eligible caregivers was
51.9% (80/154). Following the baseline assessment, the
participants were randomly allocated to either PiB or the
waiting-list control group."

"At T1, 68 participants had completed the
postintervention or postwaiting list assessment and blinding
was intact for 46% (31/68), unsuccessful for 49% (33/68), and
for 7% (5/68), a conjecture of allocation was expressed."

"It was not possible to blind the participants because of obvious differences between the interventions in content and mode of delivery."

## 11b) If relevant, description of the similarity of interventions

(this item is usually not relevant for ehealth trials as it refers to similarity of a placebo or sham intervention to a active medication/intervention)

### Does your paper address CONSORT subitem 11b? *

Copy and paste relevant sections from the manuscript (include quotes in quotation marks "like this" to indicate direct quotes from your manuscript), or elaborate on this item by providing additional information not in the ms, or briefly explain why the item is not applicable/relevant for your study

Not applicable for this study, as the control group was a waiting-list control group receiving usual care.

## 12a) Statistical methods used to compare groups for primary and secondary outcomes

NPT: When applicable, details of whether and how the clustering by care providers or centers was addressed

### Does your paper address CONSORT subitem 12a? *

Copy and paste relevant sections from the manuscript (include quotes in quotation marks "like this" to indicate direct quotes from your manuscript), or elaborate on this item by providing additional information not in the ms, or briefly explain why the item is not applicable/relevant for your study

Not applicable, this is not a cluster randomised trial.

### 12a-i) Imputation techniques to deal with attrition / missing values

Imputation techniques to deal with attrition / missing values: Not all participants will use the intervention/comparator as intended and attrition is typically high in ehealth trials. Specify how participants who did not use the application or dropped out from the trial were treated in the statistical analysis (a complete case analysis is strongly discouraged, and simple imputation techniques such as LOCF may also be problematic [4]).

| | 1 | 2 | 3 | 4 | 5 | |
|---|---|---|---|---|---|---|
| subitem not at all important | ○ | ○ | ○ | ○ | ● | essential |

## Does your paper address subitem 12a-i? *

Copy and paste relevant sections from the manuscript (include quotes in quotation marks "like this" to indicate direct quotes from your manuscript), or elaborate on this item by providing additional information not in the ms, or briefly explain why the item is not applicable/relevant for your study

"Intention-to-treat analyses was not fully possible, as intervention non-completers refused to participate in further assessments. However, we did include participants that were not completely compliant (completed only 2, 3 or no modules at all) in the analyses.42 Drop-out was higher in the intervention group compared to the control group, but selective drop-out was not demonstrated as completers did not differ from non-completers at baseline."

## 12b) Methods for additional analyses, such as subgroup analyses and adjusted analyses

## Does your paper address CONSORT subitem 12b? *

Copy and paste relevant sections from the manuscript (include quotes in quotation marks "like this" to indicate direct quotes from your manuscript), or elaborate on this item by providing additional information not in the ms, or briefly explain why the item is not applicable/relevant for your study

"To examine differences between outcomes for the intervention and the waiting-list control group during the intervention period, an analysis of covariance (ANCOVA) was conducted with outcome at post intervention as the dependent variable, intervention (Partner in Balance, waiting-list control group) as the between-subjects variable and per outcome its baseline value, age, sex, emotional instability, quality of the relationship, educational level and relationship to the care recipient as covariates. If significant, the inter-group effect size was calculated according to Cohen's d."

## X26) REB/IRB Approval and Ethical Considerations [recommended as subheading under "Methods"] (not a CONSORT item)

## X26-i) Comment on ethics committee approval

| | 1 | 2 | 3 | 4 | 5 | |
|---|---|---|---|---|---|---|
| subitem not at all important | ○ | ○ | ○ | ○ | ◉ | essential |

## Does your paper address subitem X26-i?

Copy and paste relevant sections from the manuscript (include quotes in quotation marks "like this" to indicate direct quotes from your manuscript), or elaborate on this item by providing additional information not in the ms, or briefly explain why the item is not applicable/relevant for your study

"The Medical Ethics Committee of the Maastricht University Medical Center+ (MUMC+) approved this study (#12-4-059) and the study was registered in the Dutch trial register (NTR4748). The study protocol and supporting SPIRIT checklist are available." See for the protocol paper PMID: 27142676

## x26-ii) Outline informed consent procedures

Outline informed consent procedures e.g., if consent was obtained offline or online (how? Checkbox, etc.?), and what information was provided (see 4a-ii). See [6] for some items to be included in informed consent documents.

| | 1 | 2 | 3 | 4 | 5 | |
|---|---|---|---|---|---|---|
| subitem not at all important | ○ | ○ | ○ | ○ | ◉ | essential |

## Does your paper address subitem X26-ii?

Copy and paste relevant sections from the manuscript (include quotes in quotation marks "like this" to indicate direct quotes from your manuscript), or elaborate on this item by providing additional information not in the ms, or briefly explain why the item is not applicable/relevant for your study

"The Medical Ethics Committee of the Maastricht University Medical Center+ (MUMC+) approved this study (#12-4-059) and the study was registered in the Dutch trial register (NTR4748). The study protocol and supporting SPIRIT checklist are available." See for the protocol paper PMID: 27142676

## X26-iii) Safety and security procedures

Safety and security procedures, incl. privacy considerations, and any steps taken to reduce the likelihood or detection of harm (e.g., education and training, availability of a hotline)

| | 1 | 2 | 3 | 4 | 5 | |
|---|---|---|---|---|---|---|
| subitem not at all important | ○ | ○ | ○ | ○ | ○ | essential |

## Does your paper address subitem X26-iii?

Copy and paste relevant sections from the manuscript (include quotes in quotation marks "like this" to indicate direct quotes from your manuscript), or elaborate on this item by providing additional information not in the ms, or briefly explain why the item is not applicable/relevant for your study

"The Medical Ethics Committee of the Maastricht University Medical Center+ (MUMC+) approved this study (#12-4-059) and the study was registered in the Dutch trial register (NTR4748). The study protocol and supporting SPIRIT checklist are available." See for the protocol paper PMID: 27142676

"Potential participants with insufficient cognitive abilities to engage in the online self-management program; overburdened or with severe health problems as determined by study staff; or who cared for people with dementia caused by human immunodeficiency virus (HIV), acquired brain impairment, Down syndrome, chorea associated with Huntington's disease or alcohol abuse were excluded from participation."

"The personal coaches were trained, experienced professionals (psychologists and psychiatric nurses) from one of the participating organizations. They attented a 2-hour training in self-management techniques, goal setting and online help, and regular supervision meetings."

## RESULTS

## 13a) For each group, the numbers of participants who were randomly assigned, received intended treatment, and were analysed for the primary outcome

NPT: The number of care providers or centers performing the intervention in each group and the number of patients treated by each care provider in each center

### Does your paper address CONSORT subitem 13a? *

Copy and paste relevant sections from the manuscript (include quotes in quotation marks "like this" to indicate direct quotes from your manuscript), or elaborate on this item by providing additional information not in the ms, or briefly explain why the item is not applicable/relevant for your study

"A total of 163 caregivers expressed an interest to participate. See Figure 3 for the study flowchart. Details are described elsewhere.42 Table 1 lists the baseline data for the included caregivers (N=81)."

"Table 1
Intervention (N=41) Waiting-list (N=40)"

"Table 2. Analysis of covariance comparing intervention and control group at post-test
Control (N=37) Intervention (N=31)"

## 13b) For each group, losses and exclusions after randomisation, together with reasons

## Does your paper address CONSORT subitem 13b? (NOTE: Preferably, this is shown in a CONSORT flow diagram) *

Copy and paste relevant sections from the manuscript (include quotes in quotation marks "like this" to indicate direct quotes from your manuscript), or elaborate on this item by providing additional information not in the ms, or briefly explain why the item is not applicable/relevant for your study

"A total of 163 caregivers expressed an interest to participate. See Figure 3 for the study flowchart."

Details on the recruitment procedure were described in the recently published process paper (JMIR MS #7666; PMID: 29258980)

## 13b-i) Attrition diagram

Strongly recommended: An attrition diagram (e.g., proportion of participants still logging in or using the intervention/comparator in each group plotted over time, similar to a survival curve) or other figures or tables demonstrating usage/dose/engagement.

| | 1 | 2 | 3 | 4 | 5 | |
|---|---|---|---|---|---|---|
| subitem not at all important | ○ | ○ | ● | ○ | ○ | essential |

## Does your paper address subitem 13b-i?

Copy and paste relevant sections from the manuscript or cite the figure number if applicable (include quotes in quotation marks "like this" to indicate direct quotes from your manuscript), or elaborate on this item by providing additional information not in the ms, or briefly explain why the item is not applicable/relevant for your study

This item was described in the recently published process paper (JMIR MS #7666; PMID: 29258980)

"Regarding the dose delivered, out of the program completers (n=49), 87.8% (43/49) completed all 4 modules, 6.1% (3/49) completed 3 modules, and 6.1% (3/49) completed 2 modules. The tracked usage data showed 21,946 clicks per module, including exploring the website (2444 clicks), viewing the psychological educative information (3922 clicks), completing the assignments and change plan (8748 clicks), contacting the personal coach (6489 clicks), and visiting the discussion forum (310 clicks). The total intervention time ranged from 4 to 32 weeks (mean 13.9 [SD 6.8]). Reasons for intervention period variability were holidays, illness, busy schedules, and technical difficulties. Following the intervention period, 77.6% of the program completers (38/49) requested access to the additional modules with (16/49) or without (33/49) the coach at their disposal for questions."

## 14a) Dates defining the periods of recruitment and follow-up

## Does your paper address CONSORT subitem 14a? *

Copy and paste relevant sections from the manuscript (include quotes in quotation marks "like this" to indicate direct quotes from your manuscript), or elaborate on this item by providing additional information not in the ms, or briefly explain why the item is not applicable/relevant for your study

"This randomized controlled trial was carried out between 2014 and 2016 in the Netherlands."

"From September 2014 to December 2015, family caregivers of people with (very) mild dementia of all subtypes (Clinical Dementia Rating (CDR) score 0.5-1) [28] were recruited from memory clinics (MUMC+, Elkerliek Hospital Helmond, Catharina Hospital Eindhoven) and ambulatory mental health clinics (Virenze-RIAGG Maastricht, MET ggz Roermond) in the south of the Netherlands, via caregiver support services, and via the website of the Dutch Alzheimer Association (www.alzheimernederland.nl)."

"For this study self-report data from the baseline visit (T0) and after 8 weeks (T1) were compared. These data were collected in writing by an independent research assistant who was unknown to the allocation of the treatment, separately from the coach visits."

## 14a-i) Indicate if critical “secular events” fell into the study period

Indicate if critical “secular events” fell into the study period, e.g., significant changes in Internet resources available or “changes in computer hardware or Internet delivery resources”

| | 1 | 2 | 3 | 4 | 5 | |
|---|---|---|---|---|---|---|
| subitem not at all important | ○ | ○ | ◉ | ○ | ○ | essential |

## Does your paper address subitem 14a-i?

Copy and paste relevant sections from the manuscript (include quotes in quotation marks "like this" to indicate direct quotes from your manuscript), or elaborate on this item by providing additional information not in the ms, or briefly explain why the item is not applicable/relevant for your study

not applicable for this study, no secular events took place during this time period.

## 14b) Why the trial ended or was stopped (early)

### Does your paper address CONSORT subitem 14b? *

Copy and paste relevant sections from the manuscript (include quotes in quotation marks "like this" to indicate direct quotes from your manuscript), or elaborate on this item by providing additional information not in the ms, or briefly explain why the item is not applicable/relevant for your study

not applicable, the trial was not stopped early.

## 15) A table showing baseline demographic and clinical characteristics for each group

NPT: When applicable, a description of care providers (case volume, qualification, expertise, etc.) and centers (volume) in each group

### Does your paper address CONSORT subitem 15? *

Copy and paste relevant sections from the manuscript (include quotes in quotation marks "like this" to indicate direct quotes from your manuscript), or elaborate on this item by providing additional information not in the ms, or briefly explain why the item is not applicable/relevant for your study

"Table 1 lists the baseline data for the included caregivers (N=81)."

### 15-i) Report demographics associated with digital divide issues

In ehealth trials it is particularly important to report demographics associated with digital divide issues, such as age, education, gender, social-economic status, computer/Internet/ehealth literacy of the participants, if known.

| | 1 | 2 | 3 | 4 | 5 | |
|---|---|---|---|---|---|---|
| subitem not at all important | ○ | ○ | ○ | ○ | ◉ | essential |

### Does your paper address subitem 15-i? *

Copy and paste relevant sections from the manuscript (include quotes in quotation marks "like this" to indicate direct quotes from your manuscript), or elaborate on this item by providing additional information not in the ms, or briefly explain why the item is not applicable/relevant for your study

"Table 1 lists the baseline data for the included caregivers (N=81)."

## 16) For each group, number of participants (denominator) included in each analysis and whether the analysis was by original assigned groups

### 16-i) Report multiple “denominators” and provide definitions

Report multiple “denominators” and provide definitions: Report N’s (and effect sizes) “across a range of study participation [and use] thresholds” [1], e.g., N exposed, N consented, N used more than x times, N used more than y weeks, N participants “used” the intervention/comparator at specific pre-defined time points of interest (in absolute and relative numbers per group). Always clearly define “use” of the intervention.

| | 1 | 2 | 3 | 4 | 5 | |
|---|---|---|---|---|---|---|
| subitem not at all important | ○ | ○ | ○ | ○ | ◉ | essential |

### Does your paper address subitem 16-i? *

Copy and paste relevant sections from the manuscript (include quotes in quotation marks "like this" to indicate direct quotes from your manuscript), or elaborate on this item by providing additional information not in the ms, or briefly explain why the item is not applicable/relevant for your study

"Baseline:
Intervention (N=41) Waiting-list (N=40)

Post-test:
Control (N=37) Intervention (N=31)"

This item was described in the recently published process paper (JMIR MS #7666; PMID: 29258980):

"Regarding the dose delivered, out of the program completers (n=49), 87.8% (43/49) completed all 4 modules, 6.1% (3/49) completed 3 modules, and 6.1% (3/49) completed 2 modules. The tracked usage data showed 21,946 clicks per module, including exploring the website (2444 clicks), viewing the psychological educative information (3922 clicks), completing the assignments and change plan (8748 clicks), contacting the personal coach (6489 clicks), and visiting the discussion forum (310 clicks). The total intervention time ranged from 4 to 32 weeks (mean 13.9 [SD 6.8]). Reasons for intervention period

weeks (mean [illegible] [SD [illegible]]). Reasons for intervention period variability were holidays, illness, busy schedules, and technical difficulties. Following the intervention period, 77.6% of the program completers (38/49) requested access to the additional modules with (16/49) or without (33/49) the coach at their disposal for questions."

A total of 163 caregivers were invited to participate.
See Figure 1 for the study flowchart. If they expressed interest, family caregivers (n=138) received a detailed information letter. Of the information recipients, IC was signed by 58.0% (80/138). Of the 163 recruited caregivers, 154 were eligible for participation. The participation rate of eligible caregivers was 51.9% (80/154). Following the baseline assessment, the participants were randomly allocated to either PiB or the waiting-list control group. The researcher (LMMB) not involved in the assessments performed the allocation. A research assistant blind to the allocation conducted the assessments and recorded the blinding success and reason for the possible unmasking on the case record form. At T1, 68 participants had completed the postintervention or postwaiting list assessment and blinding was intact for 46% (31/68), unsuccessful for 49% (33/68), and for 7% (5/68), a conjecture of allocation was expressed."

## 16-ii) Primary analysis should be intent-to-treat

Primary analysis should be intent-to-treat, secondary analyses could include comparing only “users”, with the appropriate caveats that this is no longer a randomized sample (see 18-i).

| | 1 | 2 | 3 | 4 | 5 | |
|---|---|---|---|---|---|---|
| subitem not at all important | ○ | ○ | ○ | ○ | ◉ | essential |

## Does your paper address subitem 16-ii?

Copy and paste relevant sections from the manuscript (include quotes in quotation marks "like this" to indicate direct quotes from your manuscript), or elaborate on this item by providing additional information not in the ms, or briefly explain why the item is not applicable/relevant for your study

"Intention-to-treat analyses was not fully possible, as intervention non-completers refused to participate in further assessments. However, we did include participants that were not completely compliant (completed only 2, 3 or no modules at all) in the analyses.42 Drop-out was higher in the intervention group compared to the control group, but selective drop-out was not demonstrated as completers did not differ from non-completers at baseline."

## 17a) For each primary and secondary outcome, results for each group, and the estimated effect size and its precision (such as 95% confidence interval)

## Does your paper address CONSORT subitem 17a? *

Copy and paste relevant sections from the manuscript (include quotes in quotation marks "like this" to indicate direct quotes from your manuscript), or elaborate on this item by providing additional information not in the ms, or briefly explain why the item is not applicable/relevant for your study

"Table 2 shows the results of the ANCOVA at T1 on self-efficacy (care management and service use), depression, mastery, perceived stress, anxiety, and quality of life. After controlling for age, sex, emotional instability, and quality of the relationship, significant effects in favor of the intervention group were found for self-efficacy care management (F(1,60)= 10.37, P=0.002, d=0.85), and self-efficacy service use (F(1,60)= 11.47, P=0.001, d=0.93), but not for depression (F(1,60)=1.13, P=0.293). Significant effects in favor of the intervention group were also demonstrated for mastery (F(1,60)= 12.66, P=0.001, d=0.94), and quality of life (F(1,60)= 4.83, P=0.032, d=0.58), but not for perceived stress (F(1,60)= 3.40, P=0.071), and anxiety (F(1,60)= 0.80, P=0.374)."

"Table 2. Analysis of covariance comparing intervention and control group at post-test
Outcome
Mean (SD/SE)
Mean (SD/SE)
Mean difference2
(95% CI)
F
d"

## 17a-i) Presentation of process outcomes such as metrics of use and intensity of use

In addition to primary/secondary (clinical) outcomes, the presentation of process outcomes such as metrics of use and intensity of use (dose, exposure) and their operational definitions is critical. This does not only refer to metrics of attrition (13-b) (often a binary variable), but also to more continuous exposure metrics such as "average session length". These must be accompanied by a technical description how a metric like a "session" is defined (e.g., timeout after idle time) [1] (report under item 6a).

| | 1 | 2 | 3 | 4 | 5 | |
|---|---|---|---|---|---|---|
| subitem not at all important | ○ | ○ | ○ | ○ | ◉ | essential |

## Does your paper address subitem 17a-i?

Copy and paste relevant sections from the manuscript (include quotes in quotation marks "like this" to indicate direct quotes from your manuscript), or elaborate on this item by providing additional information not in the ms, or briefly explain why the item is not applicable/relevant for your study

This item was described in the recently published process paper (JMIR MS #7666; PMID: 29258980):

"Regarding the dose delivered, out of the program completers (n=49), 87.8% (43/49) completed all 4 modules, 6.1% (3/49) completed 3 modules, and 6.1% (3/49) completed 2 modules. The tracked usage data showed 21,946 clicks per module, including exploring the website (2444 clicks), viewing the psychological educative information (3922 clicks), completing the assignments and change plan (8748 clicks), contacting the personal coach (6489 clicks), and visiting the discussion forum (310 clicks). The total intervention time ranged from 4 to 32 weeks (mean 13.9 [SD 6.8]). Reasons for intervention period variability were holidays, illness, busy schedules, and technical difficulties. Following the intervention period, 77.6% of the program completers (38/49) requested access to the additional modules with (16/49) or without (33/49) the coach at their disposal for questions."

## 17b) For binary outcomes, presentation of both absolute and relative effect sizes is recommended

### Does your paper address CONSORT subitem 17b? *

Copy and paste relevant sections from the manuscript (include quotes in quotation marks "like this" to indicate direct quotes from your manuscript), or elaborate on this item by providing additional information not in the ms, or briefly explain why the item is not applicable/relevant for your study

"Table 2. Analysis of covariance comparing intervention and control group at post-test
Outcome
Mean (SD/SE)
Mean (SD/SE)
Mean difference2
(95% CI)
F
d
Crude1
Adjusted2
Crude1
Adjusted2"

## 18) Results of any other analyses performed, including subgroup analyses and adjusted analyses, distinguishing pre-specified from exploratory

## Does your paper address CONSORT subitem 18? *

Copy and paste relevant sections from the manuscript (include quotes in quotation marks "like this" to indicate direct quotes from your manuscript), or elaborate on this item by providing additional information not in the ms, or briefly explain why the item is not applicable/relevant for your study

"Prior to the analysis, data was checked for missing values, outliers, and normality. Possible differences between the study groups' baseline characteristics were tested with t-tests for continuous variables and $\chi^2$-tests for categorical variables. Non-parametric tests were used when necessary in case of non-normality.
To examine differences between outcomes for the intervention and the waiting-list control group during the intervention period, an analysis of covariance (ANCOVA) was conducted with outcome at post intervention as the dependent variable, intervention (Partner in Balance, waiting-list control group) as the between-subjects variable and per outcome its baseline value, age, sex, emotional instability, quality of the relationship, educational level and relationship to the care recipient as covariates. If significant, the inter-group effect size was calculated according to Cohen's d. Effect sizes of 0.2 are considered small, 0.5 is considered medium and 0.8 high.33 IBM SPSS statistics 22.0 for Macintosh was used and all tests of significance reported mean change and were two-tailed with α set at 0.05."

## 18-i) Subgroup analysis of comparing only users

A subgroup analysis of comparing only users is not uncommon in ehealth trials, but if done, it must be stressed that this is a self-selected sample and no longer an unbiased sample from a randomized trial (see 16-iii).

| | 1 | 2 | 3 | 4 | 5 | |
|---|---|---|---|---|---|---|
| subitem not at all important | ○ | ○ | ● | ○ | ○ | essential |

## Does your paper address subitem 18-i?

Copy and paste relevant sections from the manuscript (include quotes in quotation marks "like this" to indicate direct quotes from your manuscript), or elaborate on this item by providing additional information not in the ms, or briefly explain why the item is not applicable/relevant for your study

not applicable for the present study; users were compared to a control group not receiving the intervention.

## 19) All important harms or unintended effects in each group

(for specific guidance see CONSORT for harms)

### Does your paper address CONSORT subitem 19? *

Copy and paste relevant sections from the manuscript (include quotes in quotation marks "like this" to indicate direct quotes from your manuscript), or elaborate on this item by providing additional information not in the ms, or briefly explain why the item is not applicable/relevant for your study

This item was addressed in detail in the intervention design paper published in JMIR Res Prot (JMIR Res Prot MS #5142; PMID: PMC4795319)

"Monitoring of the recruitment and execution of the study will be conducted by the trial monitoring committee of the MUMC+ (Clinical Trial Center Maastricht). Adverse events (AEs) and serious adverse events (SAEs) are not anticipated but cannot be ignored. If participants drop out, they will be asked if they had experienced an adverse or harmful event during the study period that could be attributed to “Partner in Balance.” Included participants will be asked the same question during the post-intervention assessment and at the 3-, 6- and 12-month follow-ups. All AEs and SAEs will be recorded. SAEs will be reported to the accredited Medical Ethics Committee that approved the protocol. AEs will be followed until they have abated or until a stable situation has been reached. Depending on the event, follow-up may involve additional tests or medical procedures, as indicated, and/or referral to the general physician or a medical specialist. If participants do not agree to this procedure, they cannot participate in the study.

SPIRIT checklist:
11b. Discontinuing or modifying the intervention.
Participants will be able to leave the trial, if they wish to, at any time and for any reason. An end of trial form will be completed for all trial members, detailing the reason for leaving the trial e.g. choosing to leave; illness; death; loss to follow-up. This is not a medical or pharmaceutical intervention, so we will not modify the delivery of the intervention.

22. Harms
Serious adverse events (SAEs) are not anticipated during this trial, but unanticipated adverse events are always possible. If participants drop out, they

will be asked if they had experienced an adverse or harmful event during the study period that could be attributed to ‘Partner in Balance’. Included participants will be asked the same question during the post-intervention assessment and at the 3-, 6- and 12-month follow-ups. All AEs and SAEs will be recorded.
A serious adverse event (SAE) is defined as an untoward occurrence that: (a) results in death,
(b) is life-threatening,
(c) requires hospitalization or prolongation of existing hospitalization,
(d) results in persistent or significant disability or incapacity,
(e) consists of a congenital anomaly or birth defect, or
(f) is otherwise considered medically significant by the investigator.
SAEs will be reported to the accredited MEC that approved the protocol. AEs will be followed until they have
13
Effectiveness of the blended care self-management program ‘Partner in Balance’ for early-stage dementia caregivers: study protocol for a randomized controlled trial v5 10.09.2015 SPIRIT checklist
abated or until a stable situation has been reached. Depending on the event, follow-up may involve additional tests or medical procedures, as indicated, and/or referral to the general physician or a medical specialist. If participants do not agree to this procedure, they cannot participate in the study. Reporting of SAEs will include if, in the opinion of the Principal Investigator, the event was:
• 'related': that is, it resulted from administration of any of the research procedures; and
• 'unexpected': that is, the type of event is not listed in the protocol as an expected occurrence.
Reports of related and unexpected SAEs will be submitted within 15 days of the Principal Investigator becoming aware of the event."

## 19-i) Include privacy breaches, technical problems

Include privacy breaches, technical problems. This does not only include physical “harm” to participants, but also incidents such as perceived or real privacy breaches [1], technical problems, and other unexpected/unintended incidents. “Unintended effects” also includes unintended positive effects [2].

| | 1 | 2 | 3 | 4 | 5 | |
|---|---|---|---|---|---|---|
| subitem not at all important | ○ | ○ | ○ | ○ | ◉ | essential |

## Does your paper address subitem 19-i?

Copy and paste relevant sections from the manuscript (include quotes in quotation marks "like this" to indicate direct quotes from your manuscript), or elaborate on this item by providing additional information not in the ms, or briefly explain why the item is not applicable/relevant for your study

This item was addressed in detail in the intervention design paper published in JMIR Res Prot (JMIR Res Prot MS #5142; PMID: PMC4795319) SPIRIT checklist:

"11b. Discontinuing or modifying the intervention.
Participants will be able to leave the trial, if they wish to, at any time and for any reason. An end of trial form will be completed for all trial members, detailing the reason for leaving the trial e.g. choosing to leave; illness; death; loss to follow-up. This is not a medical or pharmaceutical intervention, so we will not modify the delivery of the intervention.

22. Harms
Serious adverse events (SAEs) are not anticipated during this trial, but unanticipated adverse events are always possible. If participants drop out, they will be asked if they had experienced an adverse or harmful event during the study period that could be attributed to ‘Partner in Balance’. Included participants will be asked the same question during the post-intervention assessment and at the 3-, 6- and 12-month follow-ups. All AEs and SAEs will be recorded.
A serious adverse event (SAE) is defined as an untoward occurrence that: (a) results in death,
(b) is life-threatening,
(c) requires hospitalization or prolongation of existing hospitalization,
(d) results in persistent or significant disability or incapacity,
(e) consists of a congenital anomaly or birth defect, or
[illegible]

(f) is otherwise considered medically significant by the investigator.
SAEs will be reported to the accredited MEC that approved the protocol. AEs will be followed until they have
13
Effectiveness of the blended care self-management program ‘Partner in Balance’ for early-stage dementia caregivers: study protocol for a randomized controlled trial v5 10.09.2015 SPIRIT checklist
abated or until a stable situation has been reached. Depending on the event, follow-up may involve additional tests or medical procedures, as indicated, and/or referral to the general physician or a medical specialist. If participants do not agree to this procedure, they cannot participate in the study. Reporting of SAEs will include if, in the opinion of the Principal Investigator, the event was:
• 'related': that is, it resulted from administration of any of the research procedures; and
• 'unexpected': that is, the type of event is not listed in the protocol as an expected occurrence.
Reports of related and unexpected SAEs will be submitted within 15 days of the Principal Investigator becoming aware of the event."

"Technical issues with logging in and
communicating with the personal coach were resolved with the
team of Web experts."

## 19-ii) Include qualitative feedback from participants or observations from staff/researchers

Include qualitative feedback from participants or observations from staff/researchers, if available, on strengths and shortcomings of the application, especially if they point to unintended/unexpected effects or uses. This includes (if available) reasons for why people did or did not use the application as intended by the developers.

| | 1 | 2 | 3 | 4 | 5 | |
|---|---|---|---|---|---|---|
| subitem not at all important | ○ | ○ | ○ | ○ | ◉ | essential |

## Does your paper address subitem 19-ii?

Copy and paste relevant sections from the manuscript (include quotes in quotation marks "like this" to indicate direct quotes from your manuscript), or elaborate on this item by providing additional information not in the ms, or briefly explain why the item is not applicable/relevant for your study

This item was described in the recently published process paper (JMIR MS #7666; PMID: 29258980):

"The information on intervention quality (relevance, feasibility, and performance according to protocol) was gathered from the
perspective of both coaches and participants. Data collection
from the perspective of coaches involved the registration of
protocol deviations plus the amount and intensity of contact
with caregivers on a structured registration form (Multimedia
Appendix 1), an 8-item questionnaire rating the overall usability
of PiB and its relevance for caregivers and coaches, with 4
multiple-choice items rated on a 5-point scale (1=completely
disagree to 5=completely agree) (Multimedia Appendix 2) and
4 open-ended items on advantages, disadvantages,
recommendations for other organizations or colleagues, and
general appreciation of the program. Data from the perspective
of the participants were collected postintervention with a
semistructured interview (Textbox 1) with participants in both
the intervention and waiting-list group."

# DISCUSSION

## 22) Interpretation consistent with results, balancing benefits and harms, and considering other relevant evidence

NPT: In addition, take into account the choice of the comparator, lack of or partial blinding, and unequal expertise of care providers or centers in each group

## 22-i) Restate study questions and summarize the answers suggested by the data, starting with primary outcomes and process outcomes (use)

Restate study questions and summarize the answers suggested by the data, starting with primary outcomes and process outcomes (use).

| | 1 | 2 | 3 | 4 | 5 | |
|---|---|---|---|---|---|---|
| subitem not at all important | ○ | ○ | ○ | ○ | ◉ | essential |

## Does your paper address subitem 22-i? *

Copy and paste relevant sections from the manuscript (include quotes in quotation marks "like this" to indicate direct quotes from your manuscript), or elaborate on this item by providing additional information not in the ms, or briefly explain why the item is not applicable/relevant for your study

"This randomized controlled study evaluated the first blended care intervention for caregivers of people with early-stage dementia developed together with potential users following the MRC Framework and demonstrated a significant improvement in care management self-efficacy, service use self-efficacy, mastery and quality of life after receiving the 'Partner in Balance' intervention, compared to a waiting-list control group receiving care as usual. Effect sizes were medium (>0.5) for quality of life to high (>0.8) for self-efficacy and mastery. No differences between groups were demonstrated for caregiver depression, anxiety, and perceived stress.

Results on caregiver self-efficacy, mastery, and quality of life are in line with previous results in an uncontrolled study21 and results of previous e-health interventions for dementia caregivers.19 Furthermore, the results of the present study fit the Stress and Coping paradigm by Lazarus and Folkman34 and the Social Learning theory by Bandura7, suggesting that taking charge of the changes in one's life increases self-efficacy and general wellbeing. Learning to positively manage life with dementia instead of managing the dementia itself in a self-management program may have facilitated caregivers' adaptation to their new caregiving role. The program's focus on enhancing positive, intact experiences that are tailored to the individual caregiver's situation could explain the positive effects on caregiver self-efficacy.8"

## 22-ii) Highlight unanswered new questions, suggest future research

Highlight unanswered new questions, suggest future research.

| | 1 | 2 | 3 | 4 | 5 | |
|---|---|---|---|---|---|---|
| subitem not at all important | ○ | ○ | ○ | ○ | ◉ | essential |

## Does your paper address subitem 22-ii?

Copy and paste relevant sections from the manuscript (include quotes in quotation marks "like this" to indicate direct quotes from your manuscript), or elaborate on this item by providing additional information not in the ms, or briefly explain why the item is not applicable/relevant for your study

"However, we expected that higher levels of wellbeing or quality of life could be the result of a decrease in stress7,34, which could not be derived from the results of the present study. It is conceivable that interventions aimed at the early stages may not be capable to decrease burden and stress, as these are relatively low during the early stages6, leaving little room for improvement. Previous caregiver interventions demonstrating positive effects on burden and stress were not specifically aimed at early-stages of dementia.17,35-39 Future follow up of PiB effects could clarify if an increase in self-efficacy results in a decrease or prevention of increased stress and depression on the long term."

## 20) Trial limitations, addressing sources of potential bias, imprecision, and, if relevant, multiplicity of analyses

## 20-i) Typical limitations in ehealth trials

Typical limitations in ehealth trials: Participants in ehealth trials are rarely blinded. Ehealth trials often look at a multiplicity of outcomes, increasing risk for a Type I error. Discuss biases due to non-use of the intervention/usability issues, biases through informed consent procedures, unexpected events.

| | 1 | 2 | 3 | 4 | 5 | |
|---|---|---|---|---|---|---|
| subitem not at all important | ○ | ○ | ○ | ○ | ◉ | essential |

## Does your paper address subitem 20-i? *

Copy and paste relevant sections from the manuscript (include quotes in quotation marks "like this" to indicate direct quotes from your manuscript), or elaborate on this item by providing additional information not in the ms, or briefly explain why the item is not applicable/relevant for your study

"The waiting-list period may have affected the differences in outcomes between both groups. The effects of waiting are highly variable and depend on the characteristics of the sample and of the trial.(Hesser) However, this design allowed all potentially interested participants to participate in the intervention program, which may have increased their motivation to participate given that usual care for (very) mild dementia caregivers often either does not include counselling or includes only very infrequent counseling.41 Furthermore, the waiting-list group was not deprived of usual care. An alternative would be a pseudo-intervention in which only psycho-education or only attention of the coach is provided, but the aim of this study was not to evaluate merely the online aspect of the intervention, but the effect of the blended-care intervention of which psycho-education and face-to-face contacts are integral parts.
Intention-to-treat analyses was not fully possible, as intervention non-completers refused to participate in further assessments. However, we did include participants that were not completely compliant (completed only 2, 3 or no modules at all) in the analyses.42 Drop-out was higher in the intervention group compared to the control group, which could have resulted in inflated effect sizes. However, selective drop-out was not demonstrated as completers did not differ from non-completers at baseline. Often mentioned reasons for drop-out were no need for help or refusal by the care recipient, which was demonstrated previously as reasons of non-use of formal services.43,44 Furthermore, a higher rate of drop-out in the intervention group has previously been reported. Previous RCT’s even controlled for any possible loss of power beforehand by increasing the sample of the intervention group. Nevertheless, the current effect sizes should be interpreted with caution. Although the power of our group was not jeopardized based on our power calculation, future studies could consider controlling for a higher rate of drop-out in the intervention group to prevent loss of power. "

## 21) Generalisability (external validity, applicability) of the trial findings

NPT: External validity of the trial findings according to the intervention, comparators, patients, and care providers or centers involved in the trial

### 21-i) Generalizability to other populations

Generalizability to other populations: In particular, discuss generalizability to a general Internet population, outside of a RCT setting, and general patient population, including applicability of the study results for other organizations

| | 1 | 2 | 3 | 4 | 5 | |
|---|---|---|---|---|---|---|
| subitem not at all important | ○ | ○ | ○ | ○ | ◉ | essential |

### Does your paper address subitem 21-i?

Copy and paste relevant sections from the manuscript (include quotes in quotation marks "like this" to indicate direct quotes from your manuscript), or elaborate on this item by providing additional information not in the ms, or briefly explain why the item is not applicable/relevant for your study

"High face validity was demonstrated as the program was evaluated in multiple institutions with multiple coaches of different backgrounds. Development together with the potential users and a pilot evaluation following the MRC Framework may have increased its effectiveness."

"Our sample was not limited to memory clinics only, but the included participants may represent a subgroup of all dementia caregivers in the early stages. Caregivers in the early stages often decline formal care and it is conceivable that many were not familiar with the care parties involved in recruitment and were therefore overlooked in this study.43,44 This could have resulted in a highly motivated sample more open to support.40 Furthermore, only computer-literate caregivers could be included, which represents only around 59% of dementia caregivers.45 However, seniors’ use of Internet is expected to rise in the near future46, increasing the accessibility of PiB."

## 21-ii) Discuss if there were elements in the RCT that would be different in a routine application setting

Discuss if there were elements in the RCT that would be different in a routine application setting (e.g., prompts/reminders, more human involvement, training sessions or other co-interventions) and what impact the omission of these elements could have on use, adoption, or outcomes if the intervention is applied outside of a RCT setting.

| | 1 | 2 | 3 | 4 | 5 | |
|---|---|---|---|---|---|---|
| subitem not at all important | ○ | ○ | ○ | ○ | ◉ | essential |

## Does your paper address subitem 21-ii?

Copy and paste relevant sections from the manuscript (include quotes in quotation marks "like this" to indicate direct quotes from your manuscript), or elaborate on this item by providing additional information not in the ms, or briefly explain why the item is not applicable/relevant for your study

"High face validity was demonstrated as the program was evaluated in multiple institutions with multiple coaches of different backgrounds. Development together with the potential users and a pilot evaluation following the MRC Framework may have increased its effectiveness. "

"Our sample was not limited to memory clinics only, but the included participants may represent a subgroup of all dementia caregivers in the early stages. Caregivers in the early stages often decline formal care and it is conceivable that many were not familiar with the care parties involved in recruitment and were therefore overlooked in this study.43,44 This could have resulted in a highly motivated sample more open to support.40 Furthermore, only computer-literate caregivers could be included, which represents only around 59% of dementia caregivers.45 However, seniors' use of Internet is expected to rise in the near future46, increasing the accessibility of PiB. "

## OTHER INFORMATION

## 23) Registration number and name of trial registry

## Does your paper address CONSORT subitem 23? *

Copy and paste relevant sections from the manuscript (include quotes in quotation marks "like this" to indicate direct quotes from your manuscript), or elaborate on this item by providing additional information not in the ms, or briefly explain why the item is not applicable/relevant for your study

"Trial registration: Dutch Trial Register (NTR): NTR4748; http://www.trialregister.nl (Archived by WebCite at http://www.webcitation.org/6vSb2t9Mg)"

## 24) Where the full trial protocol can be accessed, if available

## Does your paper address CONSORT subitem 24? *

Cite a Multimedia Appendix, other reference, or copy and paste relevant sections from the manuscript (include quotes in quotation marks "like this" to indicate direct quotes from your manuscript), or elaborate on this item by providing additional information not in the ms, or briefly explain why the item is not applicable/relevant for your study

See for the protocol paper PMID: 27142676

## 25) Sources of funding and other support (such as supply of drugs), role of funders

## Does your paper address CONSORT subitem 25? *

Copy and paste relevant sections from the manuscript (include quotes in quotation marks "like this" to indicate direct quotes from your manuscript), or elaborate on this item by providing additional information not in the ms, or briefly explain why the item is not applicable/relevant for your study

"Competing interest
All authors have completed the ICMJE uniform disclosure form at www.icmje.org/coi_disclosure.pdf and declare: all authors had financial support from Maastricht University for the submitted work; no financial relationships with any organisations that might have an interest in the submitted work in the previous three years; no other relationships or activities that could appear to have influenced the submitted work."

## X27) Conflicts of Interest (not a CONSORT item)

### X27-i) State the relation of the study team towards the system being evaluated

In addition to the usual declaration of interests (financial or otherwise), also state the relation of the study team towards the system being evaluated, i.e., state if the authors/evaluators are distinct from or identical with the developers/sponsors of the intervention.

| | 1 | 2 | 3 | 4 | 5 | |
|---|---|---|---|---|---|---|
| subitem not at all important | ○ | ○ | ○ | ◉ | ○ | essential |

### Does your paper address subitem X27-i?

Copy and paste relevant sections from the manuscript (include quotes in quotation marks "like this" to indicate direct quotes from your manuscript), or elaborate on this item by providing additional information not in the ms, or briefly explain why the item is not applicable/relevant for your study

"Competing interest
All authors have completed the ICMJE uniform disclosure form at www.icmje.org/coi_disclosure.pdf and declare: all authors had financial support from Maastricht University for the submitted work; no financial relationships with any organisations that might have an interest in the submitted work in the previous three years; no other relationships or activities that could appear to have influenced the submitted work."

## About the CONSORT EHEALTH checklist

### As a result of using this checklist, did you make changes in your manuscript? *

- ○ yes, major changes
- ◉ yes, minor changes
- ○ no

## What were the most important changes you made as a result of using this checklist?

more detailed information about the randomisation procedures + informed consent

## How much time did you spend on going through the checklist INCLUDING making changes in your manuscript *

8 hours

## As a result of using this checklist, do you think your manuscript has improved? *

- (x) yes
- ( ) no
- ( ) Other:

## Would you like to become involved in the CONSORT EHEALTH group?

This would involve for example becoming involved in participating in a workshop and writing an "Explanation and Elaboration" document

- ( ) yes
- (x) no
- ( ) Other:

## Any other comments or questions on CONSORT EHEALTH

Your answer

## STOP - Save this form as PDF before you click submit

To generate a record that you filled in this form, we recommend to generate a PDF of this page (on a Mac, simply select "print" and then select "print as PDF") before you submit it.

When you submit your (revised) paper to JMIR, please upload the PDF as supplementary file.

Don't worry if some text in the textboxes is cut off, as we still have the complete information in our database. Thank you!

## Final step: Click submit !

Click submit so we have your answers in our database!

SUBMIT

Never submit passwords through Google Forms.

This content is neither created nor endorsed by Google. Report Abuse - Terms of Service - Additional Terms

Google Forms
